# Genome-Wide Analysis of *In Vivo* Binding of the Master Regulator DasR in *Streptomyces coelicolor* Identifies Novel Non-Canonical Targets

**DOI:** 10.1371/journal.pone.0122479

**Published:** 2015-04-15

**Authors:** Magdalena A. Świątek-Połatyńska, Giselda Bucca, Emma Laing, Jacob Gubbens, Fritz Titgemeyer, Colin P. Smith, Sébastien Rigali, Gilles P. van Wezel

**Affiliations:** 1 Molecular Biotechnology, Institute of Biology Leiden, Leiden University, PO Box 9502, 2300 RA Leiden, The Netherlands; 2 Department of Microbial and Cellular Sciences, Faculty of Health and Medical Sciences, University of Surrey, Guildford, Surrey GU2 7XH, United Kingdom; 3 Department of Oecotrophologie, Münster University of Applied Sciences, Corrensstr. 25, 48149 Münster, Germany; 4 Centre for Protein Engineering, Université de Liège, Institut de Chimie B6a, Sart-Tilman, B-4000 Liège, Belgium; University Paris South, FRANCE

## Abstract

Streptomycetes produce a wealth of natural products, including over half of all known antibiotics. It was previously demonstrated that N-acetylglucosamine and secondary metabolism are closely entwined in streptomycetes. Here we show that DNA recognition by the N-acetylglucosamine-responsive regulator DasR is growth-phase dependent, and that DasR can bind to sites in the *S*. *coelicolor* genome that have no obvious resemblance to previously identified DasR-responsive elements. Thus, the regulon of DasR extends well beyond what was previously predicted and includes a large number of genes with functions far removed from N-acetylglucosamine metabolism, such as genes for small RNAs and DNA transposases. Conversely, the DasR regulon during vegetative growth largely correlates to the presence of canonical DasR-responsive elements. The changes in DasR binding *in vivo* following N-acetylglucosamine induction were studied in detail and a possible molecular mechanism by which the influence of DasR is extended is discussed. Discussion of DasR binding was further informed by a parallel transcriptome analysis of the respective cultures. Evidence is provided that DasR binds directly to the promoters of all genes encoding pathway-specific regulators of antibiotic production in *S*. *coelicolor*, thereby providing an exquisitely simple link between nutritional control and secondary metabolism.

## Introduction

The ability of bacteria to adapt to a wide range of nutritional environments requires intricate regulatory systems to facilitate the key routes of primary metabolism. The flux of carbon and nitrogen is typically controlled by a number of global, pleiotropic regulators, each targetting a number of specific, non-pleiotropic regulators in their response regulon. The global regulators ensure that the flux of essential metabolites is closely monitored and tuned [1,2]. Streptomycetes are mycelial bacteria with a complex multicellular life style [3]. Adaptation to changing conditions in the environment is particularly challenging and requires a network of sensory systems, well represented in the *Streptomyces coelicolor* genome by 45 ECF (extracytoplasmic function) sigma factors genes and some 70 two-component regulatory systems [4]. As producers of over half of known antibiotics, streptomycetes are a paradigm of secondary metabolite-producing microorganisms [5,6]. Typically, antibiotic biosynthetic gene clusters are, at the very least, activated by a pathway-specific regulator within or adjacent to the cluster, and in a growth-phase dependent manner [7]. A pivotal question to address is how primary environmental signals are relayed to global carbon and nitrogen control and further to the control of development and antibiotic production. The elucidation of the *S*. *coelicolor* genome sequence—the first from an antibiotic-producing actinomycete [8]—was a landmark event, and showed that the antibiotic-producing potential of actinomycetes had been underestimated. Indeed, actinomycete genomes typically contain some 20 sets of putative biosynthetic genes for secondary metabolites [8,9,10,11], and it is yet unclear how many of these are antibiotics [12]. Many of these gene clusters are not expressed under typical laboratory conditions of rapid growth on nutrient-rich media and are often referred to as sleeping or cryptic antibiotic clusters. This may explain why they have been missed in the massive screening efforts performed by ‘Big Pharma’ [13,14]. Many antibiotic biosynthetic gene clusters are regulated in subtle ways so as to allow the organisms to adapt to life in the soil, with its varying physical, chemical and biological stresses, and the challenge is now to exploit this untapped source of potentially valuable natural products [6,15,16].

To allow activation and screening of cryptic antibiotic biosynthetic clusters, detailed insight is required into the linkages between environmental (nutritional) signals and secondary metabolite production. Carbon source utilization is a major determining factor in the metabolic control of antibiotic production [17,18]. The major control system for carbon utilization in bacteria is carbon catabolite repression (CCR). In the model organisms *Escherichia coli* and *Bacillus subtilis*, CCR is mediated via the phosphoenolpyruvate-dependent phosphotransferase system (PTS; reviewed in [19,20,21]). In streptomycetes, the PTS does not seem to play a major role in carbon control and CCR is mediated through glucose kinase, although the mechanism is still unknown [22,23]. Additionally, glucose is not internalised via the PTS but rather via the major facilitator GlcP [24]. In streptomycetes the PTS fulfills a dual role, and is involved in both *N*-acetylglucosamine (GlcNAc) uptake [25,26] as well as in the switch from normal growth to development [27]. This switch is somehow mediated via the accumulation of extracellular GlcNAc, which causes developmental arrest and blockage of antibiotic production in *S*. *coelicolor* [27,28]. GlcNAc is the monomer of the abundant natural polymer chitin—after cellulose the second most abundant carbohydrate on earth—and also a major constituent of cell-wall peptidoglycan. GlcNAc is a primary source of carbon and nitrogen for streptomycetes. The GlcNAc regulon is controlled by the GntR-family regulator DasR [27,29,30], and DNA-binding activity is inhibited *in vitro* by glucosamine-6-phosphate [27], a metabolic product derived from GlcNAc. *in silico* and *in vitro* evidence revealed that direct repression of the *actII*-ORF4 and *redZ*, the pathway-specific regulators of actinorhodin and prodiginine production, respectively [28], was likely.

Previous studies showed the involvement of DasR in the control of genes involved in GlcNAc/chitin catabolism [27,31], antibiotic production [28], siderophore biosynthesis [32] and stress response [33]. Additionally, bioinformatic predictions using the PREDetector algorithm [34] revealed some 200 sequences that conformed to the consensus binding site for DasR (*dre*, for DasR responsive element), namely the palindromic 16-bp consensus sequence A(G/C)TGGTCTAGACCA(G/C)T. These target sequences are primarily associated with genes related to primary metabolism, sugar transport and extracellular polysaccharide hydrolysis, as well as antibiotic production [32]. Moreover, evidence for the functional importance of several of these sequences was demonstrated by showing DasR binding *in vitro* and changes in the level of associated transcript(s) in a DasR knockout.

In this work we use a genome-wide analysis to determine directly the sites of DasR binding *in vivo* and correlate them with changes in gene expression. This has highlighted major and highly dynamic changes in its binding pattern both in a growth phase-dependent manner (binding during vegetative growth or sporulation) and after the exogenous addition of the ligand GlcNAc.

## Results

We previously showed that the phenotype of *dasR* null mutants and the extracellular accumulation of N-acetylglucosamine resulted in similar phenotypes, with enhanced antibiotic production and accelerated development under poor growth conditions, and blocked development and antibiotic production on rich media [28]. Linkage between the effect of GlcNAc and the phenotype of *dasR* null mutants is most likely provided by GlcN-6P, which acts as a ligand for DasR [27]. However, the differential effect of GlcNAc under different growth conditions and at different time points remained unexplained and detailed biological insights into the genome-wide distribution of DasR binding *in vivo* is therefore a prerequisite to better understand the biological role of DasR. We therefore conducted ChIP-on-chip analysis on wild-type *S*. *coelicolor* M145 carrying the integrative vector pGAM29, which expresses C-terminally 3xFLAG-tagged DasR, using the *dasR* null mutant GAM29 as the control. Two different types of genome-wide DNA-binding studies were performed; first we analysed DasR binding in cultures grown on minimal media (MM) agar plates with mannitol as the sole carbon source for the comparative analysis of DasR binding during vegetative and developmental growth, and subsequently we performed induction experiments, adding GlcNAc to liquid-grown cultures to monitor the changes on DasR binding in both rich and poor media.

### Direct control by DasR of its core regulon and of developmental genes depends on the developmental status of the cell

Firstly, we analysed DasR binding during growth on MM agar plates with mannitol as the sole carbon source, whereby two biological replicates were analysed. Samples were collected at 24 h (vegetative growth) and 54 h (sporulation), with similar growth rates and timing of development for both strains. Using a combined approach of statistical (two or more significantly enriched probes) and manual inspection (clear peaks were located by visual inspection of profile data on a genome browser), DasR binding events were identified at 21 and 51 promoter-proximal regions bound in the 24 h and 54 h old samples, respectively (Table 1 and S1 Table; rRNA operons were not taken into account). In total 10 binding events (*nagE1*, *nagE2*, *nagKA*, scr3092, *dasR-dasA* intergenic region, scr5239, tRNAGln-Glu, *ptsH*, SCO6032-6033 intergenic region and SCO7056) were shared between the 24 and 54 h samples. It should be noted at this point that we also performed eight additional ChIP-on-chip experiments on liquid-grown NMMP and R5 cultures, to analyse the effect of GlcNAc on *in vivo* binding (see below). Taken together, this provided very good validation for the *in vivo* binding of DasR, with up to eight independent verifications of the target genes (Table 1).

**Table 1:** DasR binding sites identified by ChIP-on-chip experiments and EMSAs.

Gene ID or nearest gene	Name	DasR binding (Chip-on-chip) ^a^										Total^^^	EMSA [Ref.]	*dre* element		
		24h	54h	Induction#										cis-element	position^b^	score
				MM				R5								
				T0	T1	T2	T3	T0	T1	T2	T3					
**Primary metabolism**																
SCO1390*-SCO1391*	*Crr-pts*	+	+	+	-	+	-	+	+	+	+	8	+ [27]	tgtggtctagacctct	-130	17.14
SCO1429*54h	*chiD*	+	+	-	-	-	-	+	-	-	+	4	+ [31]	actggtctagtcctcc	-96	12.17
	*chiD*	+	+	-	-	-	-	+	-	-	+	4	+ [31]	aatggtccgaaccatt	-118	7.45
SCO1444*	*chI*	+	+	-	-	-	-	-	-	-	-	2	+ [31]	actggtctagtcctct	-53	16.23
	*chI*	+	+	-	-	-	-	+	-	-	+	2	+[31]	attggtccatacctat	-75	10.22
SCO2503*54h	*chiJ*	+	+	-	-	-	-	+	-	-	+	4		Aaaggtctggaccaca	-78	12.37
	*chiJ*	+	+	-	-	-	-	+	-	-	+	4		cttggtccagacctct	-99	10.78
	*chiJ*	+	+	-	-	-	-	+	-	-	+	4		tctggaccacagcact	-73	7.29
SCO2833*	chb	+	+	+	-	+	-	+	-	+	-	6		acatgtccataccaaa	-110	9.34
	*Chb*	+	+	+	-	+	-	+	-	+	-	6		gcaggtctagaccaag	-70	8.67
SCO2906	*nagE1*	+	+	-	-	-	-	+	+	+	+	6		actggtctacaccagt	-41	17.52
	*nagE1*	+	+	-	-	-	-	+	+	+	+	6		atcggtctgcaccagt	718	8.37
	*nagE1*	+	+	-	-	-	-	+	+	+	+	6		caaggtgtagacctct	1287	12.27
SCO2907	*nagE2*	+	+	-	-	-	-	+	-	+	-	4		acaggtctacaccact	-49	16.57
	*nagE2*	+	+	-	-	-	-	+	-	+	-	4		agtggtgtagaccacc	-32	14.37
	*nagE2*	+	+	-	-	-	-	+	-	+	-	4		caaggtgtagacctct	-236	12.27
SCO4284-4285-4286	*nagKA*	+	+	+	-	-	-	+	+	+	+	7	+ [30]	agaggtctagtccact	-83	16.64
	*nagKA*	+	+	+	-	-	-	+	+	+	+	7	+ [30]	ggtggtgtagacctta	-101	9.64
	SCO4286	+	+	+	-	-	-	+	+	+	+	7		agaggtctagtccact	-81	16.64
	SCO4286	+	+	+	-	-	-	+	+	+	+	7		ggtggtgtagacctta	-63	9.64
SCO5003-SCO5004*54h	*chiA*	+	+	+	-	-	-	+	-	-	-	4		ggtggtccagaccaat	-77	10.43
		+	+	+	-	-	-	+	-	-	-	4		ggtggtccagaccaat	-258	10.43
SCO5230		+	+	+	-	+	-	+	-	-	-	5		tctggtctagtcctgg	-118	9.87
SCO5231-5232*	*dasR*	+	+	+	-	+	-	+	-	-	-	5	+ [27]	cttggtctagtccata	-150	9.05
	*dasR*	+	+	+	-	+	-	+	-	-	-	5	+ [27]	tctggtctagtcctgg	755	9.87
	*dasA*	+	+	+	-	+	-	+	-	-	-	5	+ [27, 30]	actggtctacaccatt	-106	17.13
	*dasA*	+	+	+	-	+	-	+	-	-	-	5	+ [27, 30]	cttggtctagtccata	-322	9.05
SCO5236	*nagB*	+	-	-	-	-	-	+	-	-	+	3	+ [27, 30]	tgtggtttagaccaat	-68	17.51
SCO5376	*chiC*	+	-	-	-	-	-	-	-	-	-	1		ataggtctggaccaat	-109	11.92
	*chiC*	+	-	-	-	-	-	-	-	-	-	1		aaaggtctggaccata	-88	9.23
SCO5673*	*chiB*	+	+	+	+	+	+	+	-	-	-	7		attggtctggaccaaa	-63	10.69
SCO5841	*ptsH*	+	+	-	-	-	-	-	-	-	-	2	+ [27]	agttgtctagaccagt	-51	18.11
	*ptsH*	+	+	-	-	-	-	-	-	-	-	2	+ [27]	tcttgtctagaccagt	-66	16.25
SCO6004-SCO6005* ^54h^ intergenic region		-	+	+	+	+	+	+	-	-	+	7		agtggactatacctgt	-244	16.05
	*ngcE*	-	+	+	+	+	+	+	-	-	+	7		agtggactatacctgt	-334	16.05
SCO6012-SCO6013	*chiH*	+	-	-	-	-	-	+	-	-	-	2	+ [31]	aatggtctggaccaga	-111	12
	*chiH*	+	-	-	-	-	-	+	-	-	-	2	+ [31]	atgggactagaccaat	-127	10.22
		+	-	-	-	-	-	+	-	-	-	2		aatggtctggaccaga	-274	12
		+	-	-	-	-	-	+	-	-	-	2		atgggactagaccaat	-258	10.22
SCO6032-33		+	+	-	-	-	-	+	-	-	+	4		cttggtctagtccatt	-154	10
		+	+	-	-	-	-	+	-	-	+	4		cttggtctagtccatt	-278	10
SCO6300		+	-	-	-	-	-	+	-	-	-	2		ataggtctagacaaaa	-131	13.72
		+	-	-	-	-	-	+	-	-	-	2		agaggtctagacaaaa	-116	13.48
SCO6344-45		+	-	-	-	-	-	+	-	-	-	2	+ [31]	taaggtctagacctgc	-133	9.6
		+	-	-	-	-	-	+	-	-	-	2	+ [31]	gtaggtctagacctgc	-153	8.13
	*chi*	+	-	-	-	-	-	+	-	-	-	2		taaggtctagacctgc	-114	9.6
	*chi*	+	-	-	-	-	-	+	-	-	-	2		gtaggtctagacctgc	-94	8.13
SCO7225* ^54h^	*chi*	+	+	-	-	-	-	+	-	-	-	3	+ [31]	tcaggtctagacctgt	-34	15.28
	*chi*	+	+	-	-	-	-	+	-	-	-	3	+ [31]	ccttgtctagaccaat	-168	13.38
	*chi*	+	+	-	-	-	-	+	-	-	-	3	+ [31]	tatggtctagacctga	-55	13.25
SCO7335^c^	*pep1B*	-	+	-	-	-	-	-	-	-	-	1				
**Development and secondary metabolism**																
SCO3230^c^	*cdaPSI*	-	+	+	-	+	-	+	-	-	+	5				
SCO3231^c^ ^,^ * ^54h^	*cdaPSII*	-	+	+	+	-	-	+	-	-	+	5				
SCO5085	*actII-4*	-	-	+	-	+	-	+	-	-	+	4	+ [28]	tgttgagtaggcctgt	-59	10.49
SCO5877	*redD*	-	-	+	+	+	-	+	-	-	-	4		actgctggagaccggt	612	7.15
SCO5881^c^	*redZ*	-	-	+	-	+	-	+	-	-	+	4	+ [28]	agtggtttccacctca	-201	12.44
SCO6273^c^	*cpkC*	-	+	+	+	-	+	-	-	-	-	4		acatgcgtaatcaact	-13	9.2
SCO6274^c^	*cpkB*	-	+	+	+	-	+	-	-	-	-	4				
SCO6275^c^ ^,^ * ^54h^	*cpkA*	-	+	-	-	-	-	-	-	-	-	1				
SCO1276	*sigJ*	-	+	-	-	-	-	-	-	-	-	1				
SCO1488-89* ^54h^	*pyrR*	-	+	+	+	+	-	+	+	+	+	8				
	*bldD*	-	+	+	+	+	-	+	+	+	+	8				
SCO3323* ^54h^	*bldN*	-	+	-	-	-	-	-	-	-	-	1				
**Translation**																
SCO2504-05	*glyS*	-	+	-	-	-	-	-	-	-	-	1		agtggtctgcacctgg	515	10.75
SCO3092		+	+	-	-	-	-	-	+	+	+	5				
SCO3679		+	+	-	-	-	-	-	-	-	-	2		tgttgtctagtccaat	-314	15.32
SCO4092		-	-	-	-	-	-	+	+	+	+	4				
SCO5239		+	+	-	-	-	-	-	-	-	-	2	+, this paper	agtggtctagtccaca	-335	16.23
SCO5550^d^		+	+	+	-	+	-	+	+	+	+	8	+, not shown	actggtctaaaccaca	-18	17.27
SCO4123^d^		-	+	+	-	+	-	-	+	+	+	6				
SCO1792^d^		-	+	+	-	+	-	-	+	+	+	6				
SCO1390		-	+	+	-	+	-	-	+	+	+	6				
SCO5746		-	+	+	-	+	-	-	+	+	+	6				
SCO3334		-	+	+	-	+	-	-	+	+	+	6				
**Transposases and DNA topology**																
SCO0020^c^		-	+	-	-	-	-	-	-	-	-	1				
SCO0098-SCO0099		-	-	+	-	+	-	-	-	-	-	2				
SCO0567		-	-	+	+	+	-	-	-	-	-	3				
SCO1471		-	-	+	-	-	-	-	-	-	-	1				
SCO1603		-	-	+	+	+	-	-	-	-	-	3				
SCO3466		-	-	+	+	+	-	+	-	-	+	5				
SCO3467		-	-	+	+	+	-	-	-	-	-	3				
SCO3468		-	+	+	+	+	-	+	-	-	+	6				
SCO3490^c^		-	+	-	-	-	-	-	-	-	-	1		aatcgtcaagacctgt	-118	8.75
SCO4344		-	+	-	-	-	-	-	-	-	-	1				
SCO4698^c^		-	+	-	-	-	-	-	-	-	-	1				
SCO4699^c^		-	+	-	-	-	-	-	-	-	-	1		agaggtcgacacccgt	2130	9.17
SCO6400^c^		-	+	+	+	+	-	+	-	-	+	6				
SCO6627^c^	*pglX*	-	+	-	-	-	-	-	-	-	-	1		cacggtgtagacatca	841	7.41
SCO6910-SCO6911		-	-	+	+	+	-	+	-	-	+	5				
SCO7798^c^		-	+	-	-	-	-	-	-	-	-	1				
**Other**																
SCO1606		-	-	+	+	+	+	-	-	-	+	5				
SCO2078		-	+	+	+	+	+	-	-	-	+	6				
SCO3262^c^		-	+	+	+	-	-	-	-	-	+	4		acaggaggacaccatt	-118	8.53
SCO5190	*wblC*	-	-	+	+	+	+	-	-	-	-	4				
SCO5423	*pyk2*	-	-	+	+	+	+	-	-	-	-	4				
SCO7056		+	+	-	-	-	-	+	-	-	-	3		attggtctaaaccagc	-79	15.65
SCO4646	*secE*	-	-	-	-	-	-	+	+	+	+	4				
SCO4067	*dnaZ*	-	-	-	-	-	-	-	+	+	+	3				

* one probe; *54h one probe at 54h

**^a,^** +, binding;-, no binding

**^b,^** relative to the start of the gene

**^c,^** coding;

**^d,^** downstream

**^#^** Refers to loss of DasR binding following addition of GlcNAc; see S2 Table for details.

**^^^** nr of independent ChIP-chip experiments showing DasR binding

**Fig 1:**
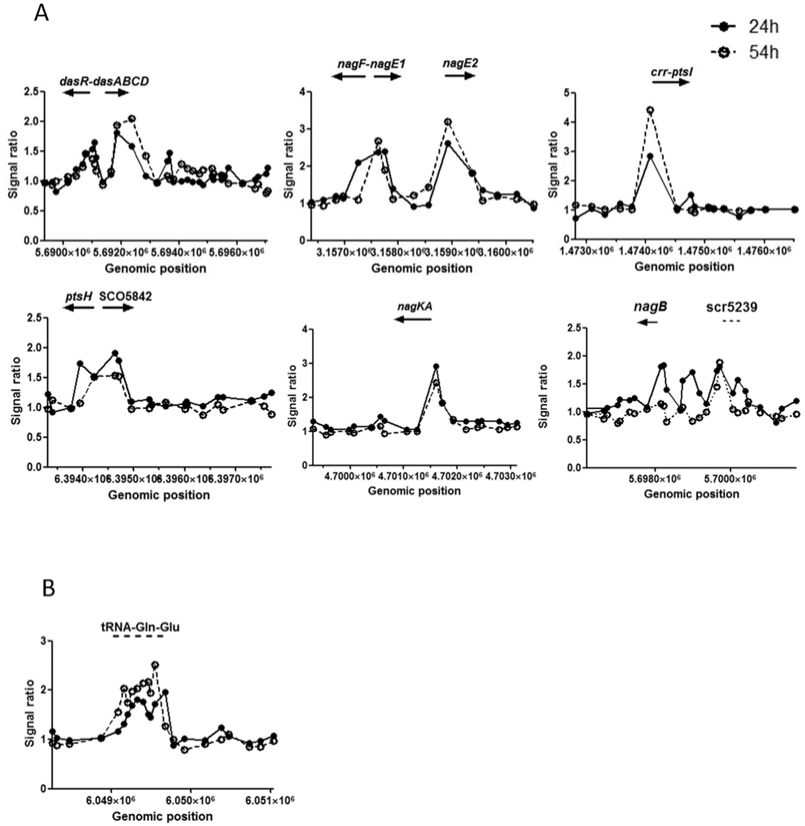
Selected DasR targets identified in ChIP-on-chip time-course experiments. Peaks indicate the presence of DasR binding sites in the regions near the *dasR*, *dasABC*, *nag* and *pts* genes and the gene cluster specifying tRNA^Gln/Glu^. Plots present DasR binding at 24 h (solid line, closed circles) and 54 h (dashed line, open circles). The arrows indicate the orientation of the target genes. Note that the y-axis (signal ratio) varies between the plots.

As expected, clear enrichments for DasR-binding were found in the upstream regions of many genes that belong to the *nag* regulon, the *pts* genes and the chitinolytic system, which can be described as the core regulon for DasR [27,28], and coincide with bioinformatically predicted *dre* elements (using the PREDetector algorithm [34]) with generally high scores (Table 1). These include the upstream regions for the *nag* metabolic genes *nagKA* and *nagB*, the *pts* genes *nagE1*, *nagE2*, *ptsH*, and members of the chitinolytic system, namely *chiA*, *chiC*, *chiD*, *chiH*, *chiJ*, SCO6032 (for *β-N*-acetylhexosaminidase), SCO6300 (for a secreted *β-N*-acetyl-glucosaminidase), SCO6345, the SCO6005-6007 operon (the SCO6004-SCO6005 intergenic region has only one probe significantly enriched), *dasA* and *dasR* itself (Fig 1 and S1 Fig show targets with two or more significantly enriched probes). The previously experimentally verified DasR targets *crr-ptsI*, *chiI*, *chiB* and *chb* [27,30,31] also came out as hits, but only had one probe significantly enriched, and therefore failed to meet our rigorous statistics (see Experimental Procedures).

**Fig 2:**
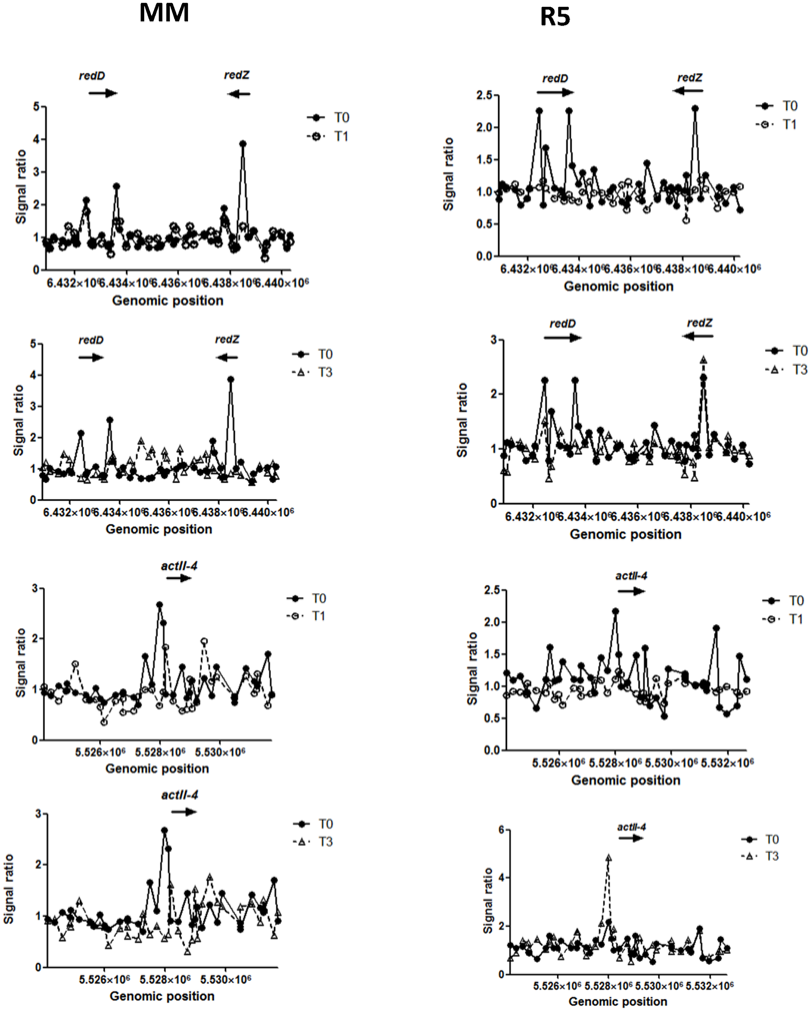
Chip-on-chip data for selected DasR targets detected in the GlcNAc induction experiment. Samples were collected prior to (T_0_, closed circles) and 30 (T_1_, open circles), 60 (T_2_) or 120 min (T_3_, open triangle) after GlcNAc addition. Plots indicate change in affinity of DasR for the promoter regions of the pathway-specific activator genes for the biosynthesis of actinorhodin (*actII-*ORF4) and prodiginine (*redD*, *redZ*) upon addition of GlcNAc (25 mM) to liquid-grown NMMP (left panel) or R5 (right panel) cultures. Arrows indicate the orientation of the targeted genes. Note that the y-axis (signal ratio) varies between the plots.

Surprisingly, the genomic distribution of the observed DasR binding showed major divergence between the cultures harvested at 24 h and 54 h, which highlights growth-phase dependent differences in the DasR-binding pattern. In particular, all of the genes of the core regulon (*nag*, *ptsH*, *chi*) that were bound by DasR at 24 h were either not bound after 54 h of growth, or (in the case of the chitinolytic genes *chiC*, *chiH*, SCO6300, SCO6345 and SCO7225) showed reduced binding. From the perspective of developmental control, only after 54 h did we see binding of DasR to any of the genes involved in development and secondary metabolism, as well as many other targets outside the core regulon. Binding was observed to the intragenic regions of three biosynthetic genes (SCO6273-6275) of the *cpk* gene cluster for the cryptic type I polyketide synthase (Cpk) and of *cdaPSI and cdaPSII* (SCO3230 and SCO3231) encoding the peptide synthetases I and II for the synthesis of calcium-dependent antibiotic (CDA). Additionally, in liquid-grown cultures binding of DasR was detected in the promoter proximal region of *actII*-ORF4 and *redD*, *redZ*, encoding the pathway-specific activator genes for Act and Red biosynthetic clusters, respectively (see Table 1, Fig 2 and below). *dre* elements were identified *in silico* for *actII*-ORF4, *redZ*, and *cpkC*, and *actII*-ORF4 and *redZ* were also corroborated *in vitro* by EMSAs.

DasR binding was observed for the promoter regions of many genes for transfer-RNA (tRNA) and noncoding RNA (ncRNA). As for ncRNAs, binding was observed to *dre* elements upstream of scr3092 [35] and scr5239 [36] (Table 1). scr5239 decreases the production of the antibiotic actinorhodin, and represses expression of the extracellular agarase gene *dagA* (SCO3471) at the post-transcriptional level [36]. Additionally, DasR likely controls the expression of several tRNA genes, as suggested by binding to multiple sequences associated with highly conserved *dre* elements—and in several independent ChIP-on-chip experiments—within the five-membered tRNA^Gln/Glu^ gene cluster, to a gene for tRNA^Gly^ upstream of SCO3679, to tRNA^Met^ and to the tRNA^Glu/Asp/Phe^ gene cluster upstream of SCO4092. Additionally, after 54 h of growth on MM agar plates, DasR binding was observed upstream of SCO2504 (*glyS*) encoding glycyl-tRNA synthetase (Table 1). Finally, binding was also observed to rRNA operons. *S*. *coelicolor* has six rRNA operons [37] and binding to them was observed previously for HspR [38]. To validate binding of DasR to scr5239, we tested if indeed DasR could bind specifically to a probe encompassing the upstream region of scr5239. Phylogenic analysis of upstream sequences revealed a strongly conserved *dre* element upstream of scr5239 in streptomycetes, with core sequence 5’-TGGTCTAGTCCA, spaced 49 nt away from the start of scr5239 (S2 Fig). EMSAs using purified His_6_-tagged DasR showed that indeed the protein bound *in vitro* to a double-stranded oligonucleotide encompassing the *dre* element, with equal efficiency as to the positive control (a fragment of the same length encompassing the *dre* element of *dasA*). The negative control (a fragment of the *bla* gene for a beta-lactamase) was not bound by DasR.

### GlcNAc-mediated alteration of DasR binding *in vivo*

Having established DasR binding during normal growth, we then wondered how *in vivo* DasR binding would respond to the exogenous addition of GlcNAc. In particular, we sought to investigate the molecular basis for the differential effect of GlcNAc addition under feast (rich media) and famine (poor media) conditions, with block or stimulation of development and antibiotic production, respectively. To address this important question, we performed ChIP-on-chip experiments for DasR following the extracellular addition of GlcNAc. For this purpose, mycelia of *S*. *coelicolor* M145 harbouring pGAM29 and the *dasR* mutant were each grown in liquid NMMP mannitol or R5 cultures, and cells were induced by the addition of 50 mM GlcNAc. Samples were collected prior to (T_0_) and 30 (T_1_), 60 (T_2_) or 120 min (T_3_) after GlcNAc addition, and DasR-enriched chromatin was analysed as described in the Experimental Procedures section. Prior to GlcNAc induction (T_0_) high ChIP-enrichment ratios demonstrated DasR binding at 72 and 66 sites in NMMP- and R5-grown mycelia, respectively (S2 Table). In line with the experiments performed on mycelia from surface-grown cultures, these sites included its core regulon *pts*, *nag* and *chi* (S3 Fig).

Interestingly, after GlcNAc addition to NMMP-grown mycelia, the DasR protein dissociated from these target sites, providing direct evidence that indeed DasR responds to the addition of GlcNAc *in vivo*, thereby reducing its affinity for its target DNA. This also provided the first *in vivo* evidence for the previously proposed signaling cascade [28] from extracellular accumulation of GlcNAc to the onset of antibiotic production via the GlcNAc-mediated release of DasR from promoters of: (i) genes for GlcNAc transport and phosphorylation by the PTS (*crr-ptsI* and *nagE2*); (ii) the transcription unit that includes *nagA* which is necessary to provide the DasR effector GlcN-6P; and (iii) *actII*-ORF4 and of *redD* and *redZ*, encoding the pathway-specific activator genes for Act and Red biosynthetic clusters, respectively, as well as from the coding sequences of *cdaPSI* and *cdaPSII* (SCO3230-3231) for CDA peptide synthetases I and II, and for *cpkBC* (for Cpk polyketide synthases; [39]). Furthermore, this also presents the first evidence that in fact DasR directly binds to genes belonging to *all* of the known antibiotic biosynthetic clusters of *S*. *coelicolor*.

In the above mentioned DasR-mediated GlcNAc signaling cascade from nutrient availability to antibiotic production, internalization via the *nagE2* product is the first step, while control of pathway-specific regulators is the last step. We previously showed that DasR and AtrA have antagonistic actions towards *nagE2* and *actII*-ORF4. AtrA is required for actinorhodin production via the direct activation of *actII*-ORF4 [40], and also activates the GlcNAc transporter gene *nagE2* [26], while DasR does the opposite. Interestingly, our data show that in fact DasR binds to the upstream region of *atrA*, and that this binding is reduced by the addition of GlcNAc to NMMP-grown mycelia and relieved when GlcNAc was added to R5 cultures. Thus, AtrA and DasR antagonise each other in terms of their influence on this important signaling cascade.

Finally, over 30 cases were found in nutrient rich R5-grown cultures where DasR binding was not relieved by the addition of GlcNAc, of which nearly 20 in fact showed enhanced binding. Examples of constant binding include *pyrR-bldD*, the upstream regions of the tRNA genes/operons tRNA^Glu/Asp/Phe^, tRNA^Gln/Glu^ and tRNA^Met^, and *secE* for the preprotein translocase subunit SecE (S4 Fig). Examples of enhanced binding of DasR include the upstream regions of *dnaZ* for DNA polymerase II subunit gamma, of *cydA* for cytochrome oxidase subunit I, of the cell division-related gene SCO2078 and of the ncRNA, scr3092 (S5 Fig).

### Transcriptomic analysis of the *dasR* null mutant

The ChIP-on-chip experiments provided insight into the global DasR binding. To obtain complementary insight into the global changes at the transcriptional level following the deletion of *dasR*, microarray analysis was performed on wild-type and *dasR* mutant strains grown on MM mannitol agar plates. Under these standard growth conditions both the wild-type and mutant displayed similar development [27]. RNA was isolated from mycelia harvested after 24 h (vegetative growth), 30 h (early aerial growth), 36 h (early aerial growth), 42 h (late aerial growth) and 54 h of growth (sporulation). Microarray analysis revealed some 1200 genes that were significantly differentially expressed in the *dasR* null mutant of *S*. *coelicolor* relative to the parent M145, and the transcription of over 400 genes was more than two-fold up- or down-regulated at one or more time points in the *dasR* mutant (across two biological replicates). Some 100 genes showed more than three-fold and 26 genes showed more than five-fold change in transcription relative to the parental strain (S3 Table). Classes of genes that were significantly differentially expressed in the *dasR* strain compared to wt related to: primary metabolism; development and secondary metabolism; SapB, chaplins, rodlins (development-related peptides) and small peptides with unknown functions; transposases and DNA recombination. The most notable changes are discussed below; for details see S3 Table.

**Fig 3:**
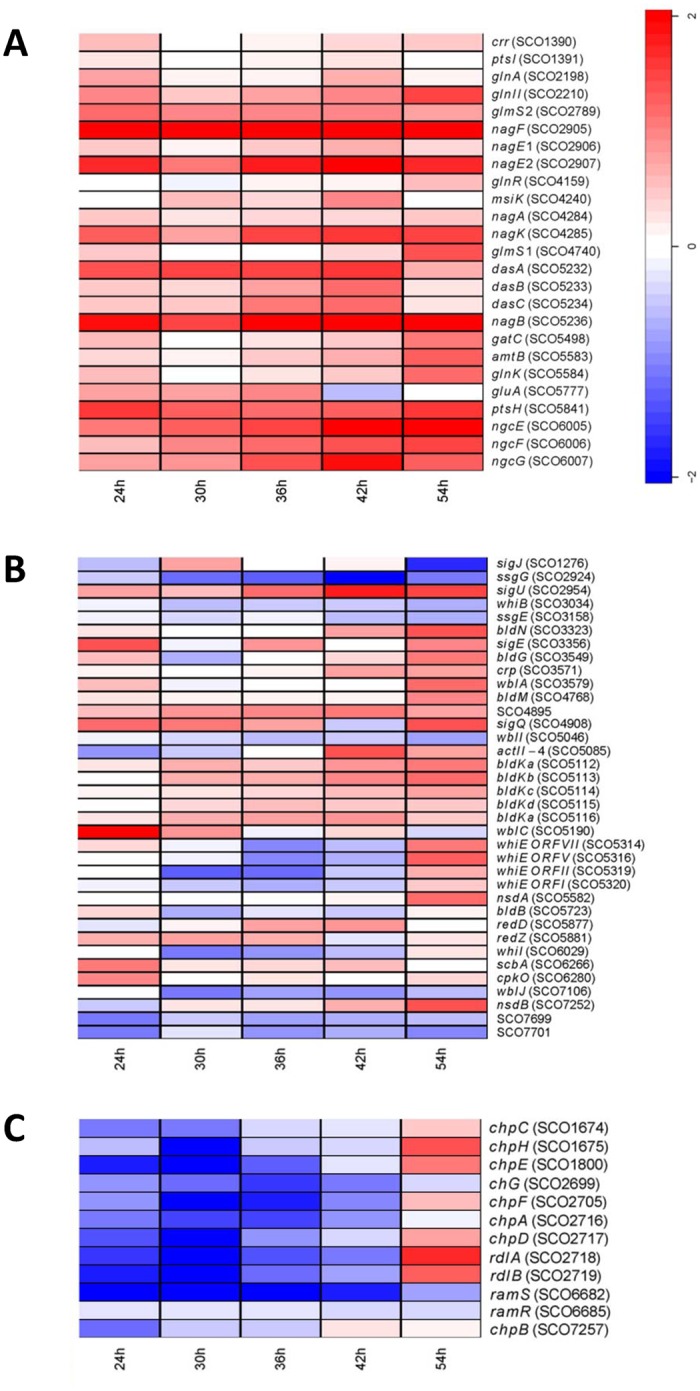
Selected categories of genes significantly differentially expressed in the *dasR* null mutant. Important categories of genes whose transcription was altered significantly in microarray experiments when comparing the *dasR* mutant GAM29 to its parent *S*. *coelicolor* M145 are shown; these are (A) genes for amino acid and (amino-)sugar transport and metabolism; (B) genes related to development and antibiotic production; (C) genes for chaplins, rodlins and SapB. RNA was isolated from *S*. *coelicolor* M145 or its *dasR* mutant GAM29 cultivated on MM mannitol agar plates after 24 h (vegetative growth), 30 h (vegetative/aerial growth), 36 h (aerial growth), 42 h (aerial growth/sporulation) and 54 h (sporulation). A heat map is provided as reference for the fold changes in log scale (log_2_(mutant/wild-type)).Note that many genes shown a larger than four-fold change in transcription. Some key genes that were experimentally validated DasR targets but with <2-fold change are also presented. For exact values see S3 Table.

Genes of the DasR core regulon were up-regulated in the *dasR* mutant and mostly at all time points, including *nagE2*, *nagF*, *ptsH (*encoding PTS components*); nagB*, *nagK* (encoding GlcNAc metabolic enzymes)*; glmS1* and *glmS2* (opposite catalytic activity as *nagB*); and *dasA-dasBC* (chitobiose transporter [41,42]) (Fig 3A). Additionally, several genes involved in central metabolism, such as glycolysis, the TCA cycle, gluconeogenesis and pyruvate metabolism, as well as for biosynthetic pathways for amino acids, nucleotides and fatty acids were up-regulated in the *dasR* mutant (S3 Table). The ABC transporter operons, in particular the branched-chain amino acid transporter operon SCO2008-SCO2012, the SCO6005-SCO6007 operon (Fig 3A) that is orthologous to the *ngcEFG* chitobiose/GlcNAc transport operon in *S*. *olivaceoviridis* [43], and some of the genes in the oligopeptide transporter operon SCO5476-SCO5480, were strongly up-regulated in the *dasR* mutant (S3 Table).

As expected, on the basis of the direct binding of DasR as observed by ChIP-on-chip analysis, transcription of some of the genes for the biosynthesis of actinorhodin (SCO5070-SCO5091) and prodiginines (SCO5877-SCO5898) was enhanced in the *dasR* mutant (S3 Table). However, while changes for many *act* and *red* genes were statistically relevant, the majority did not meet the criterion of a minimum of two-fold change in expression. Transcription of *scbA* (SCO6266) for γ-butyrolactone synthase implicated in the control of the antibiotic clusters *act*, *red* and *cpk* in *S*. *coelicolor* [44], and *cpkO* (SCO6280), for the transcriptional activator of the *cpk* cluster, was up-regulated at 24 h, followed by enhanced transcription of the *cpk* cluster in the following time point (30 h) (Fig 3B and S3 Table). Other antibiotic-related genes whose transcription was upregulated were *nsdA* (SCO5582), a negative regulator of antibiotic production in *S*. *coelicolor* [45] and its paralogue *nsdB* (SCO7252) [46]. Several genes of the deoxysugar gene cluster SCO0381-SCO0401, which shows similarity to the *ste* gene cluster of *Streptomyces* sp. 139 required for Ebosin biosynthesis, were also up-regulated in the *dasR* mutant (S3 Table). Ebosin is an exopolysaccharide (EPS) which shows anti-rheumatic arthritis activity *in vivo* [47]. It is interesting to note that in *Streptomyces* sp. 139 the *dasR* gene lies immediately adjacent to the orthologous cluster [47].

The DasR regulon includes a large number of putative transcriptional regulators, with around 40 regulatory genes at least two-fold up- or down-regulated in the mutant at one or more time points, which forms a likely explanation for the large number of differentially expressed genes in the mutant. Several early developmental genes were up-regulated in the *dasR* mutant at later time points, namely *bldK*, *bldM*, *bldN*, *crp* and *wblA* (Fig 3B), while expression of sporulation genes (*e*.*g*. *whi*, *ssg*) was left unchanged. The pentacistronic *bldK* operon encodes an oligopeptide transporter required for early stages of development [48,49], *bldN* encodes a developmentally regulated σ factor, σ^BldN^, required for aerial mycelium formation, which in turn is required for the transcription of the response regulator gene *bldM* [50], *crp* is involved in the control of spore germination [51,52], and the WhiB-like protein WblA represses antibiotic production and may also be involved in the transition from early aerial hyphal cells to the subapical stem and apical compartments that precede sporulation [53]. Some of the most highly up-regulated genes were *wblC* (SCO5190) and its flanking genes SCO5189 and SCO5191.

Strikingly, *chpABCDEFGH*, *rdlAB* and *ramS*, encoding chaplins, rodlins and SapB, respectively, were very strongly down-regulated during early time points. The chaplins and rodlins are hydrophobic ‘coat’ proteins that assemble into a rodlet layer that provides surface hydrophobicity to aerial hyphae and spores, thus allowing them to break through the moist soil surface [54,55]; the spore-associated protein SapB is a lantibiotic-type signaling peptide required for the onset of development [56]. Several of these genes featured among the 10 most strongly down-regulated genes during early time points (Fig 3C).

Finally, transcription of two other gene clusters encoding small (60–100 aa) secreted proteins and a GntR regulator [57], was also strongly repressed, with transcription of the SCO3982-SCO3988 cluster so strongly enhanced that it suggests that the cluster is completely silenced in wild-type cells by DasR (S3 Table). This gene cluster is most likely directly controlled by DasR, because the *gntR* regulatory gene SCO3986, encoding the likely regulator of this cluster, was identified as a DasR target in the ChIP-on-chip experiments on MM agar plates with mannitol at 54h (S1 Table). Having said that, binding of DasR upstream of SCO3986 was not detected at 24 h, despite that also during vegetative growth the SCO3982-SCO3988 cluster is strongly up-regulated. The regulation of the cluster therefore requires further investigation. Transcription of the paralogous gene cluster SCO3263-3268 (with SCO3982 similar to SCO3268 and so on) was also repressed by DasR S3 Table. The function of the gene clusters is yet unknown, but their strong up-regulation in the *dasR* mutant is intriguing.

## Discussion

### DasR has two classes of direct targets and binds in a development-dependent manner

Streptomycetes usually live in an environmental context of poor nitrogen and rich carbon availability [58]. In this respect, GlcNAc is a highly appropriate energy source for these bacteria, as it can be used as both a nitrogen and a carbon source. The core of the DasR regulon is formed by genes related to amino sugar metabolism, namely the *nag* and *pts* genes for GlcNAc metabolism and transport, respectively, and the *chi* regulon involved in the catabolism of polymers of GlcNAc. Since Fructose-6P, acetate and ammonia are end products of GlcNAc catabolism, the DasR core regulon is immediately connected to major metabolic pathways such as glycolysis, nucleic acids, nitrogen, and fatty acid metabolism, the tricarboxylic acid (TCA) cycle and cell-wall biosynthesis. There is an excellent correlation between the presence of a *dre* motif and DasR binding *in vitro* and *in vivo*, and indeed we have never observed a predicted *dre* with a high statistical score that is not bound by DasR *in vitro*. There were however a few examples of known core target genes that did not display ChIP-enrichment peaks across multiple samples (*e*.*g*. binding to *crr-ptsI* was only seen once); false negatives are not uncommon in ChIP-based studies and may reflect cases where the DasR is physically hidden within the cross-linked chromatin (*e*.*g*. encompassed by looped DNA and/or other transcription factors). A notable example in this context is the failure to detect a ChIP-enrichment peak for CRP bound to the *E*. *coli lac* operon promoter [59].

Novel functional categories of Class I targets (*i*.*e*. preceded by *dre* elements) identified here include genes for ncRNAs and tRNAs. Additionally, we here provide evidence that in *S*. *coelicolor* all of the known antibiotic biosynthetic gene clusters are directly controlled by DasR, namely *act*, *red*, *cpk* and *cda* for actinorhodin, undecylprodigiosin, a cryptic polyketide and calcium-dependent antibiotic, respectively. Most of these are associated with *dre* elements. However, many binding events, referred to as ‘Class II’ targets, were identified *in vivo* that are not associated with a canonical *dre* element. Some of these were identified as highly-enriched probes in as many as eight biologically independent ChIP-on-chip experiments, providing a strong case that these are indeed *bona fide* binding sites. Genome-wide analysis of the regulons of several other globally acting bacterial transcription factors show similar duality, with binding to both their cognate (consensus) target sequences and to non-canonical sites *in vivo*, even though the latter binding sites show no similarity to the consensus sequence and they are not bound *in vitro*. Other examples include LexA [60], Crp [61] and FNR [62] in *E*. *coli*, Spo0A in *Bacillus subtilis* [63], CtrA in *Caulobacter crescentus* [64] and notably also Crp [65], GlnR [66] and PhoP [67] in *Streptomyces*. For *B*. *subtilis* Spo0A, some 15% of the total number of binding sites was not bound *in vitro* [63]. AdpA of *Streptomyces griseus* and PhoP of *S*. *coelicolor* represent examples of master regulators in streptomycetes with a significant proportion of putative target genes without recognisable consensus binding sites [67,68]. It is likely that binding by these regulators to non-canonical sites requires additional factors, such as cooperative interaction with other proteins and/or changes in DNA conformation. An example of cooperative binding that is required for target recognition is that for CRP in *E*. *coli*, which binds to a noncanonical site in the *melAB* promoter in concert with MelR associated with adjacent target sites [69].

The conventional Class I targets are generally bound more avidly or, in a stochastic binding model, more frequently than the unconventional Class II targets, as the most enriched probes in the ChIP-on-chip experiments were typically those for Class I targets. This is similar to the situation observed for LexA in *E*. *coli*, where unconventional targets were also not bound by LexA *in vitro*, and bound less tightly *in vivo* [60]. A proteomics-based approach is currently being employed to elucidate how DasR binds to Class II targets *in vivo*, with particular focus on identifying possible protein partners that bind cooperatively with DasR.

The *in vivo* DNA-binding experiments revealed major differences in the binding pattern between cultures harvested at 24 h and 54 h, which highlights the rather expected growth-phase dependence of DasR binding. The notion that the affinity of a certain regulator for its binding site changes during growth is a well-accepted principle, but a growth-phase dependent change in binding, with other sites bound at different developmental stages, is to the best of our knowledge a novel concept. All of the genes of the core regulon (*nag*, *pts*, *chi*) were bound by DasR at 24 h, but after 54 h, using the same detection methods, no evident binding was found for *chiC*, *chiH*, SCO6300, SCO6345 and SCO7225. Conversely, DasR binding to genes involved development and antibiotic production (and other processes) was only found after 54 h. The pleiotropic role of DasR in the control of antibiotic production at least in *S*. *coelicolor* was further underlined by the binding of DasR to the promoter-proximal regions of *actII*-ORF4, *redD* and *redZ*, encoding the activator genes for Act and Red biosynthetic clusters, respectively [28].

### The DasR response regulon and multi-level control of its target genes

The core regulon of the DasR homologue NagR in *Bacillus* also revolves around GlcNAc metabolism, but in contrast to *dre* elements, the NagR-responsive elements are found scarcely (up to three sites per genome [70]). Comparative genomics analysis suggests that similar small DasR regulons are found in other low G+C Gram-positive bacteria such as *Streptococcus* and *Listeria* (not shown). Our work shows that streptomycetes have adopted the GlcNAc regulatory system to build a much more intricate system that controls a wide range of genes. More than 1,200 genes were significantly differentially expressed in the *dasR* null mutant of *S*. *coelicolor* (*i*.*e*. 15% of the genome) and transcription of over 400 genes altered more than two-fold, which indicates the large changes in gene expression resulting from the absence of the metabolic master regulator DasR. In a recent environmental study, microarray data on the *dasR* null mutant in soil-grown cultures in the presence of chitin revealed some 700 genes that were differentially expressed [33]. In general, deletion of developmental regulators has a major impact on gene expression in *Streptomyces*, exemplified by the more than 1000 significantly differentially expressed genes in mutants lacking the morphogene *ssgA*, which controls processes relating to cell-wall remodeling such as germination, tip growth, branching and cell division [71,72], or the very large response regulon of BldD, which directly controls a large number of developmental genes including some 40 regulatory genes [73].

The DasR regulon incorporates around 40 transcriptional regulatory genes, suggesting an extensive secondary response. Global transcriptional regulators provide an additional layer of control on top of the control mediated by specific activators and/or repressors [1]. Therefore, an ‘all or nothing’ change in transcription following deletion of *dasR* was not anticipated. The specific environmental conditions are an important parameter for the response of the regulon, and additional control systems operate, such as substrate induction for induction of sugar utilization operons, or growth phase-dependent control of antibiotic production. The additional requirement of substrate induction is exemplified by the fact that despite DasR binding to almost all of the genes of the *chi* regulon, transcription of the genes was not notably changed in *dasR* mutant cells due to the lack of chitin as inducer. Indeed, strong induction of the chitinolytic system by chitin was observed in soil-grown *S*. *coelicolor* cultures, as well as enhanced antibiotic production [33,74]. The expression levels of all these genes were up-regulated by chitin in soil cultures. This suggests that there are other mechanism(s) that overrule the inhibitory effect of DasR on gene expression.

Antibiotic production is typically extensively controlled, which ensures correct growth phase-dependent expression. Taking *actII*-ORF4 as an example, this gene is controlled by more than 10 transcriptional regulators. Besides DasR these include among others ActII-ORF4, AfsR, AtrA, SCO6008 and the stringent response mediated through ppGpp during growth cessation ([40,75,76,77]; reviewed in [7,78]). As mentioned above, our genome-wide DNA-binding experiments demonstrated that DasR directly controls gene expression of the biosynthetic pathways for Act, Red, CDA and Cpk biosynthesis and also that this control is effectively relieved by the addition of GlcNAc. This surprising control of all antibiotics produced by *S*. *coelicolor* M145 (which lacks the plasmid-borne methylenomycin biosynthetic cluster) may have evolved to ensure global control of natural product formation that is modulated by changes in nutritional conditions. The relatively small changes in gene expression of the antibiotic-related genes seen under the chosen experimental conditions are a consequence of the choice of media, which only allow minor induction of antibiotic production, but were chosen here to allow proper comparison between wild type and mutant, which requires similar (and synchronized) growth and development; however, major changes in antibiotic production are seen under conditions of feast (rich media) and famine (MM with agar as the sole carbon source) [28].

### *In vivo* response of DasR to the induction by GlcNAc

We have previously shown that glucosamine-6P (GlcN-6P) acts as an effector of DasR *in vitro* [27]. This is not unexpected, as GlcN-6P stands at the crossroads of (GlcNAc)_n_ degradation, GlcNAc transport and intracellular metabolism, glycolysis, nitrogen and lipid metabolism, as well as peptidoglycan synthesis [79,80], and many genes of these pathways are subject to control by DasR. ChIP-on-chip analysis of MM- and R5-grown cultures before and after the addition of GlcNAc revealed the response of DasR to this inducer *in vivo*. The relief of DasR binding to its core regulon following the addition of GlcNAc to cultures provides *in vivo* evidence that DasR binding indeed undergoes major changes following binding of GlcNAc-derived metabolites. In terms of antibiotic production, the same relief of binding was seen for the pathway-specific activator genes *redD* and *redZ* for prodiginine production, and *actII*-ORF4 and *atrA* for actinorhodin production, as well as biosynthetic genes for the cryptic polyketide Cpk and the calcium-dependent antibiotic CDA. This provides experimental *in vivo* proof that the activation of antibiotic production by GlcNAc is directly mediated through DasR in *S*. *coelicolor* and also shows that this goes further than the control of *actII*-ORF4 and *redZ* (which were previously identified as direct targets *in vitro* [28]).

### Connection to other developmental regulons

Our data indicate that DasR exerts developmental control by targeting the expression of a number of key regulatory genes for the control of early developmental processes, including *bldKc*, *bldM*, *bldN*, *wblA*, *wblC* and *crp*. Of these, *bldKc*, *bldN* and *wblC* were identified as direct targets by ChIP-on-chip analysis, forming Class II targets as none of them is preceded by a *dre* element. Of these, binding to *bldN* was only seen for one experiment, and care has therefore to be taken as to whether this reflects true binding. Interestingly, however, the *bldN* gene product σ^*bldN*^ is required for transcription of the *chp*, *rdl* and *ramS* genes [81], and the observed increase in *bldN* transcription in *dasR* null mutants apparently connects to the observed increase in *chp*, *rdl* and *ramS* expression during sporulation.

In terms of cross-talk with other developmental regulators, AtrA and DasR apparently counteract each other, with activating and repressing activity, respectively, on *nagE2* and *actII*-ORF4. Their gene products form core members in the signalling cascade from GlcNAc accumulation outside the cell to the activation of actinorhodin production, with NagE2 importing the inducer and ActII-ORF4 as terminal activator. In contrast, there was surprisingly little overlap between the highly pleiotropic regulatory networks of BldD and DasR. Regulators of later sporulation events do not feature among the direct DasR targets, while, conversely, the primary BldD regulon contains a plethora of such developmental regulatory genes [73], suggesting that these two proteins act in different time (and perhaps also spatial) domains. Our data suggest that *bldD* may be a Class II target for DasR, with binding upstream of *bldD* verified independently in samples obtained after 54 h of growth on MM agar plates as well as from liquid-grown R5 cultures. Transcription of *bldD* was not changed in the *dasR* deletion mutant on MM mannitol, but under these conditions both the *bldD* and the *dasR* mutants sporulate. Whether and if so when DasR controls *bldD* transcription awaits further analysis.

**Fig 4:**
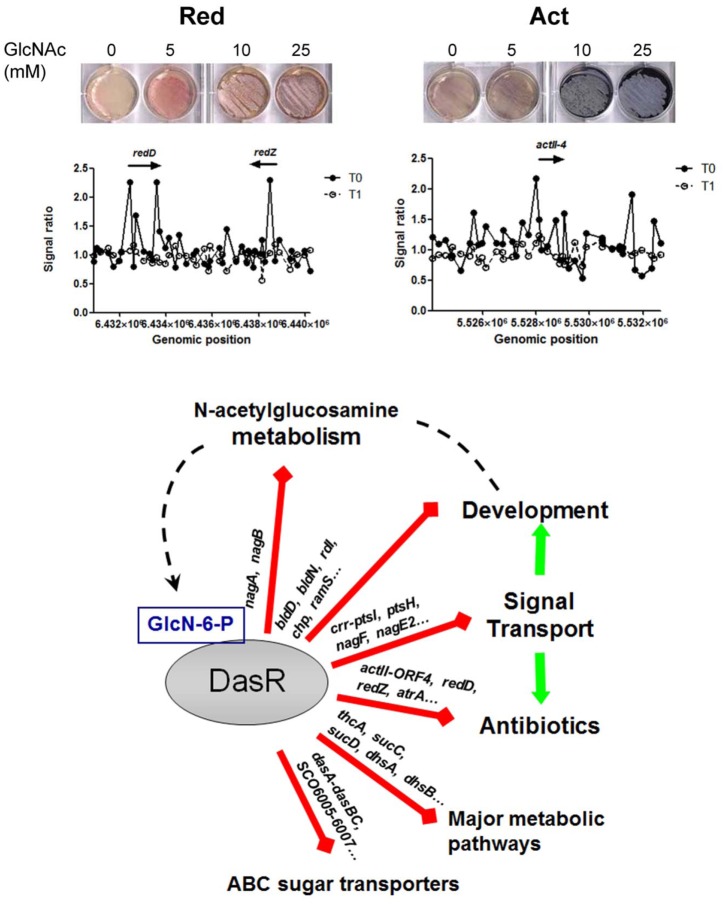
Model of the DasR regulon and its induction by *N*-acetylglucosamine. **Top:** induction of development (grey pigment) and Red (left; red pigment) or Act (right; blue pigment) production on MM agar plates with increasing concentrations of *N*-acetylglucosamine; the *S*. *coelicolor actII*-ORF4 (M510) and *redD* (M511) mutants [76] were used to demonstrate the production of only the red-pigmented undecylprodigiosin or the blue-pigmented actinorhodin, respectively. **Middle,** corresponding ChIP-chip experiments showing relief of DasR binding from the *act* and *red* regulatory genes *in vivo* after the addition of GlcNAc (examples taken from Fig 2). T_0_ (closed circles) and T_1_ (open circles), time points before and 30 min after the addition of 25 mM GlcNAc; respectively. **Bottom,** summary of the DasR-controlled processes and major targets that are directly or indirectly controlled by DasR. Red lines indicate direct binding and transcriptional repression of the target genes by DasR (for some developmental genes only based on ChIP-on-chip data). Derepression of control by DasR in response to the accumulation of GlcN-6P results in activation (green lines) of morphological development and antibiotic production, among other processes.

In conclusion, our work provides detailed insights into how DasR links nutritional control to antibiotic production and development and shows how its affinity for different binding sites changes *in vivo* in response to the extracellular signalling nutrient GlcNAc (Fig 4). The canonical binding of DasR to Class I targets is observed both *in vivo* and *in vitro*, while the unconventional binding to Class II targets presumably requires cooperative binding with another protein(s), which has hitherto not been identified. Alternatively, these sites may require conformational changes in the DNA, or even a combination of the two. DasR mediates a higher order level of control, and relief of, or induction of, binding to its target sites does not necessarily result in an immediate change in gene expression. For that, the environmental conditions are an important determining factor—such as the presence of chitin for induction of *chi* gene expression—and further experimental data need to be obtained from genome-wide studies under different growth conditions. For example, in approaches to activate poorly expressed (or even dormant) antibiotic biosynthetic genes, relief of DasR-binding may well be an important step, but better understanding of the triggers that must be in place to effectively activate their biosynthetic gene clusters should aid their discovery and exploitation.

## Experimental procedures

### Bacterial strains and growth conditions

*E*. *coli* JM109 [82] and ET12567 [83] were used for routine cloning procedures and for extracting non-methylated DNA, respectively. *S*. *coelicolor* M145 was obtained from the John Innes Centre strain collection and is the parent for the *dasR* null mutant GAM29, which had its entire coding region replaced by the apramycin resistance cassette as described previously [27]. All media and routine *Streptomyces* techniques are described in the *Streptomyces* manual [83]. R5 (regeneration media) is a rich media with glucose as carbon source and high concentration of sucrose (10% w/v) to allow protoplast regeneration, while NMMP is a standard minimal media buffered with phosphate. Cells of *E*. *coli* were grown in Luria–Bertani broth (LB) at 37°C. SFM (mannitol soya flour) agar plates were used to prepare spore suspensions. Strains were cultivated on MM agar plates with mannitol (1% w/v) (covered with cellophane discs) or in liquid R5 or NMMP supplemented with mannitol (1% w/v). For induction experiments, liquid cultures were grown at 30°C until mid-logarithmic phase (OD_600_ approximately 0.4 for NMMP cultures and 0.8 for R5 cultures), followed by the addition of GlcNAc (50 mM). For the ChIP-on-chip studies on MM mannitol solid medium mycelium was harvested after 24 h and 54 h.

### Plasmids and constructs

For the ChIP-on-chip studies *a* recombinant *dasR* gene was synthesised that allows production of DasR containing a C-terminal triple FLAG-tag epitope (GenScript, La Jolla, USA). The DNA fragment contained the *dasR* gene along with 306 bp promoter region, with the stop codon of the gene replaced by the sequence 5’-ATGGACTACAAGGACCACGACGGCGACTACAAGGACCACGACATCGACTACAAGGACGACGACGACAAGTAGACCAGAGCCCGCTCACCCGGCCCCAGATTGCGGTTGAAGTCC-3’ (stop codon underlined). In this way, the DasR protein was extended with the amino acid sequence MDYKDHDGDYKDHDIDYKDDDDK. The fragment was cloned into the integrative vector pSET152 [84]. This resulted in vector pGAM29, which expresses DasR-3xFLAG for use in ChIP-on-chip experiments.

### Electrophoretic Mobility shift assays

N-terminally His_6_-tagged DasR was overexpressed in *E*. *coli* BL21 (DE3) as described previously [27]. Proteins were isolated using Ni-NTA chromatography as described [85] and peak fractions containing the purified proteins were dialysed against modified storage buffer (50 mM Tris-HCl (pH 8.0), 50 mM KCl, 0.5 mM EDTA, 1 μM β-mercaptoethanol, 10% glycerol) and stored at -80°C. EMSAs were performed on PCR-amplified DNA as described previously [27].

### Chromatin immunoprecipitation, gDNA labeling and array hybridizations

*S*. *coelicolor* M145 containing the integrative vector pGAM29 expressing 3xFLAG-tagged DasR and the *dasR* null mutant GAM29 were grown in duplicate (see *Bacterial strains and growth conditions*). Cultures were treated with formaldehyde (final concentration 1%, 20 min, 30°C) in order to crosslink proteins to DNA. Subsequently glycine (final concentration of 0.5M) was added to quench the formaldehyde and cultures were incubated for further 5 minutes at 30°C. Mycelium was harvested by centrifugation, washed twice with 1xPBS and stored at -70°C. Chromatin isolation, labeling and hybridization were performed as described previously [38,86]. Biological replicates of DasR-3FLAG-IP chromatin were labeled with either Cy3 or Cy5—dCTP in a dye swap experimental design and co-hybridised with the mock ‘no-antibody’ IP chromatin on 4 × 44K whole genome *Streptomyces* arrays manufactured by Agilent Technologies [38].

### RNA isolation, cDNA synthesis and labeling

Total RNA was purified using the Kirby-mix protocol [83]. DNaseI treatment was used to fully remove any traces of DNA. Before use, the RNA preparations were checked for their quality and integrity on the Agilent 2100 Bioanalyzer (Agilent Technologies). cDNA synthesis and labeling was performed as previously described [38,86].

### Microarray expression data and ChIP-on-chip data analysis

For the time-course gene expression studies *S*. *coelicolor* M145 and *dasR* mutant (GAM29) were cultivated on MM mannitol agar plates overlaid with cellophane discs. Samples were taken at 24 h, 30 h, 36 h, 42 h and 54 h. Two biological replicates were analysed for all samples and for each strain. cDNA incorporating Cy3 dCTP was synthesized as described in http://www.surrey.ac.uk/fhms/microarrays/Downloads/Protocols/Strep_hyb_protocol_1005.pdf, from 10 μg of total RNA extracted from each time point. Labeled cDNA was co-hybridized with genomic DNA, used as a common reference, labeled with Cy5-dCTP as described in http://www.surrey.ac.uk/fhms/microarrays/Downloads/Protocols/Strep_hyb_protocol_1005.pdf. The fluorescently labeled samples were hybridized onto whole genome custom *Streptomyces* microarrays 4 x 44K as described in ref.35. For the ChIP-chip experiment, chromatin samples were either immuno-precipitated using a monoclonal anti-FLAG tag M2 antibody (Sigma cat. Number F1804)(sample) or incubated in absence of antibody (mock IP control). When mycelium from solid medium was used in the ChIP-chip experiment, the mycelium was harvested quickly with a spatula and immersed in 1% formaldeyde solution for 20 min at 30°C before adding glycine to 0.5 M final concentration to quench the formaldeyde. In the case of liquid cultures, the cells were treated with formaldeyde by adding the reagent directly to the culture at a final concentration of 1% and continuing the incubation for 20 min before adding the glycine as quencher. After the formaldeyde treatment the protocol followed was as described in [38].

Expression array data were normalized using the Limma package [87]. Briefly, data were normalized within each array using the global median value and then normalized across all arrays using the ‘scale’ function. The filtered data sets were then analysed using Rank Products analysis [88] via the web-based implementation of Rank-Prod [89] called RankProdIt [90]. Differentially expressed genes were identified as having a pfp (probability of false prediction) less than or equal to 0.15, equal to a false discovery rate of approximately 15%; this represents a previously validated threshold for gene expression analysis [91]. Some additional genes were also identified that demonstrated a >1.5 fold change in expression between wild-type and the *dasR* mutant. ChIP-on-chip data, allowing identification of genes/regions directly bound by DasR were analysed essentially as described previously [38]; the arrays were ‘across-array’ normalised only (using the Limma package) and poor quality probes (determined by Agilent Feature Extraction software version 9.1) were removed from downstream analysis. Probes with significant enrichment for DasR at a particular time-point were identified using a Mann-Whitney t-test to compare the log_2_ (WT-DasR antibody/mock-antibody) and log_2_ (Mutant-DasR antibody/mock-antibody) ratios for each time-point (using the R package ‘multtest’). A probe was scored as significant if the associated t-statistic was greater than 2 which, given the number of replicates in this study, corresponds to a false discovery rate of less than 10%; this was considered to be a suitable initial threshold given our application of additional subsequent thresholds and manual inspection. Additional thresholds applied to highlight putative DasR binding events for manual inspection were: (1) a log_2_ (WT-DasR antibody/mock-antibody)—log_2_ (Mutant-DasR antibody/mock-antibody) enrichment ratio of >0.3, where 0.3 is the point at which the distribution of these WT vs Mutant ratios depart from the typical Gaussian curve; (2) the presence of two significantly enriched DasR-ChIP probes within a 3.5 kb window. These DasR ChIP-enrichment regions containing putative DasR binding sites were then manually inspected to identify associated genes. Here, clear peaks within the 3.5 kb region were located by visual inspection of profile data using a genome browser.

## Supporting Information

S1 Fig Chip-on-chip data for selected DasR targets detected in time-course experiment. (PDF)

S2 Fig DasR binds the *dre* upstream of scr5239. (PDF)

S3 Fig Chip-on-chip data for selected targets for which DasR binding was relieved or reduced after GlcNAc induction. (PDF)

S4 Fig Chip-on-chip data for selected targets for which DasR binding was unchanged in response to GlcNAc induction. (PDF)

S5 Fig Chip-on-chip data for selected targets for which DasR binding was enhanced in response to GlcNAc induction. (PDF)

S1 Table Binding by DasR as identified by genome-wide ChIP-on-chip experiments. Expression, trend in microarray analysis.
(PDF)

S2 Table DasR binding before and after addition of GlcNAc to liquid-grown MM and R5 cultures. (PDF)

S3 Table Genes whose expression was altered more than two-fold in the dasR null mutant as determined by microarray experiments. (PDF)
