# Supplementary material for: Genome-Wide Analysis of In Vivo Binding of the Master Regulator DasR in Streptomyces coelicolor Identifies Novel Non-Canonical Targets
Source: PLoS One. 2015 Apr 15;10(4):e0122479. doi: 10.1371/journal.pone.0122479 (PMC4398421; doi:10.1371/journal.pone.0122479)

S1 Fig. Chip-on-chip data for selected DasR targets detected in time-course experiment. Peaks indicate the presence of DasR binding sites in the promoter regions of *chi* genes. Plots presents DasR binding at 24h (solid line, close circles) and 54h (dashed line, open circles). The arrows indicate the orientation of the target gene.

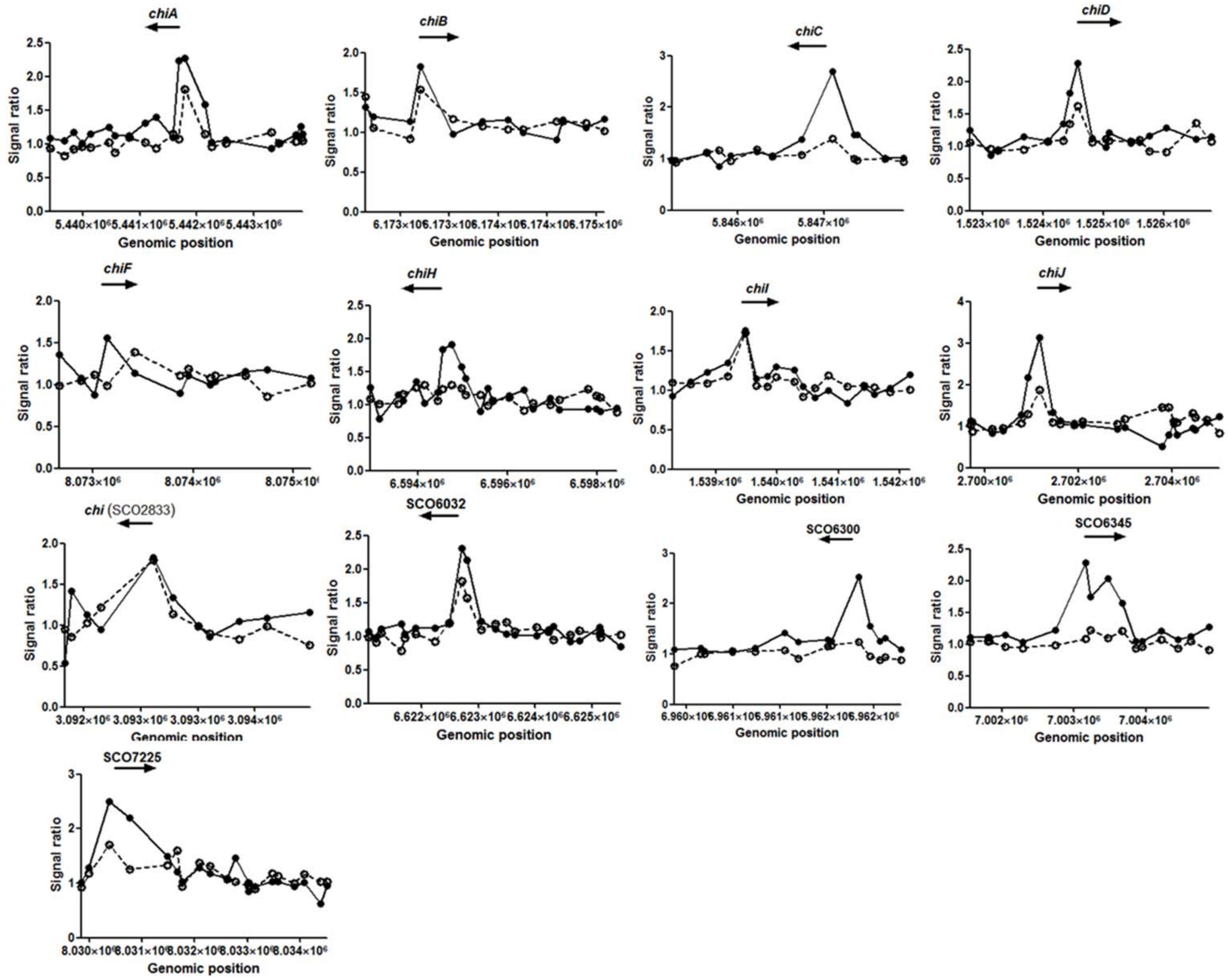

Supplement: S1 Fig — (PDF) [file pone.0122479.s001.pdf]
