# Supplementary material for: Genome-Wide Analysis of In Vivo Binding of the Master Regulator DasR in Streptomyces coelicolor Identifies Novel Non-Canonical Targets
Source: PLoS One. 2015 Apr 15;10(4):e0122479. doi: 10.1371/journal.pone.0122479 (PMC4398421; doi:10.1371/journal.pone.0122479)

S2 Fig. DasR binds the *dre* upstream of scr5239. EMSA performed with pure DasR-His and the *dre*^{scr5239}. *dre* usptream of *dasA* (*dre*^{dasA}) was used as positive control for DasR bound sequence (Rigali et al., 2004) and the *cis*-acting element of BlaI (*blaP* OP1) of *Bacillis licheniformis* was used as negative control (DNA sequence with no *dre*). EMSA were performed with 15 pmol of Cy5-labeled *dre*^{5239} probe (fp, free probe) and increasing concentrations of pure DasR-His_6 (50, 75, 100, 125, and 150 pmol, respectively) .

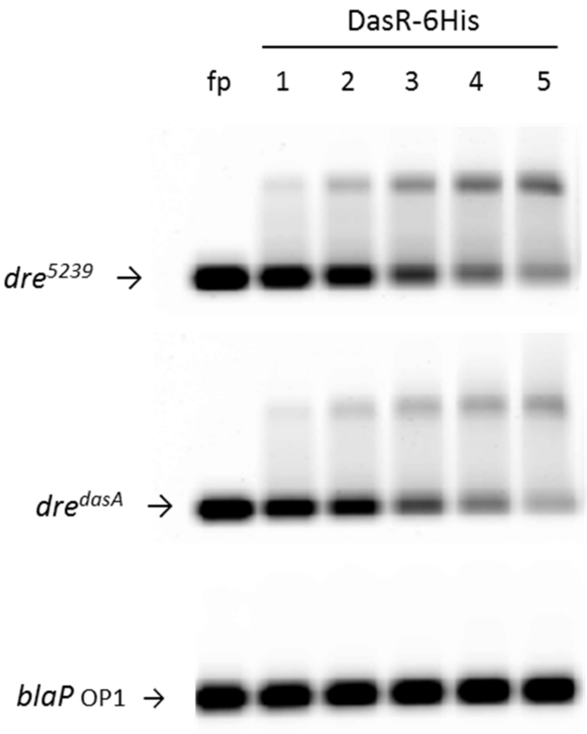

Supplement: S2 Fig — (PDF) [file pone.0122479.s002.pdf]
