# Supplementary material for: Genome-Wide Analysis of In Vivo Binding of the Master Regulator DasR in Streptomyces coelicolor Identifies Novel Non-Canonical Targets
Source: PLoS One. 2015 Apr 15;10(4):e0122479. doi: 10.1371/journal.pone.0122479 (PMC4398421; doi:10.1371/journal.pone.0122479)

S3 Fig. Chip-on-chip data for selected targets for which DasR binding was relieved or reduced after GlcNAc induction. Samples were collected prior to (T_0, closed circles) and 30 (T_1, open circles), 60 (T_2, closed triangle) or 120 min (T_3, open triangle) after GlcNAc addition. Plots indicate changing of the DasR binding to the promoter regions of 10 known DasR targets (*crr-ptsI, nagE1-nagE2, nagKA, dasR-dasABC, nagB, ptsH, pitE, chiH*, SCO6032, SCO6300) upon amino sugar addition to MM and R5. The arrows indicate the orientation of the target gene.

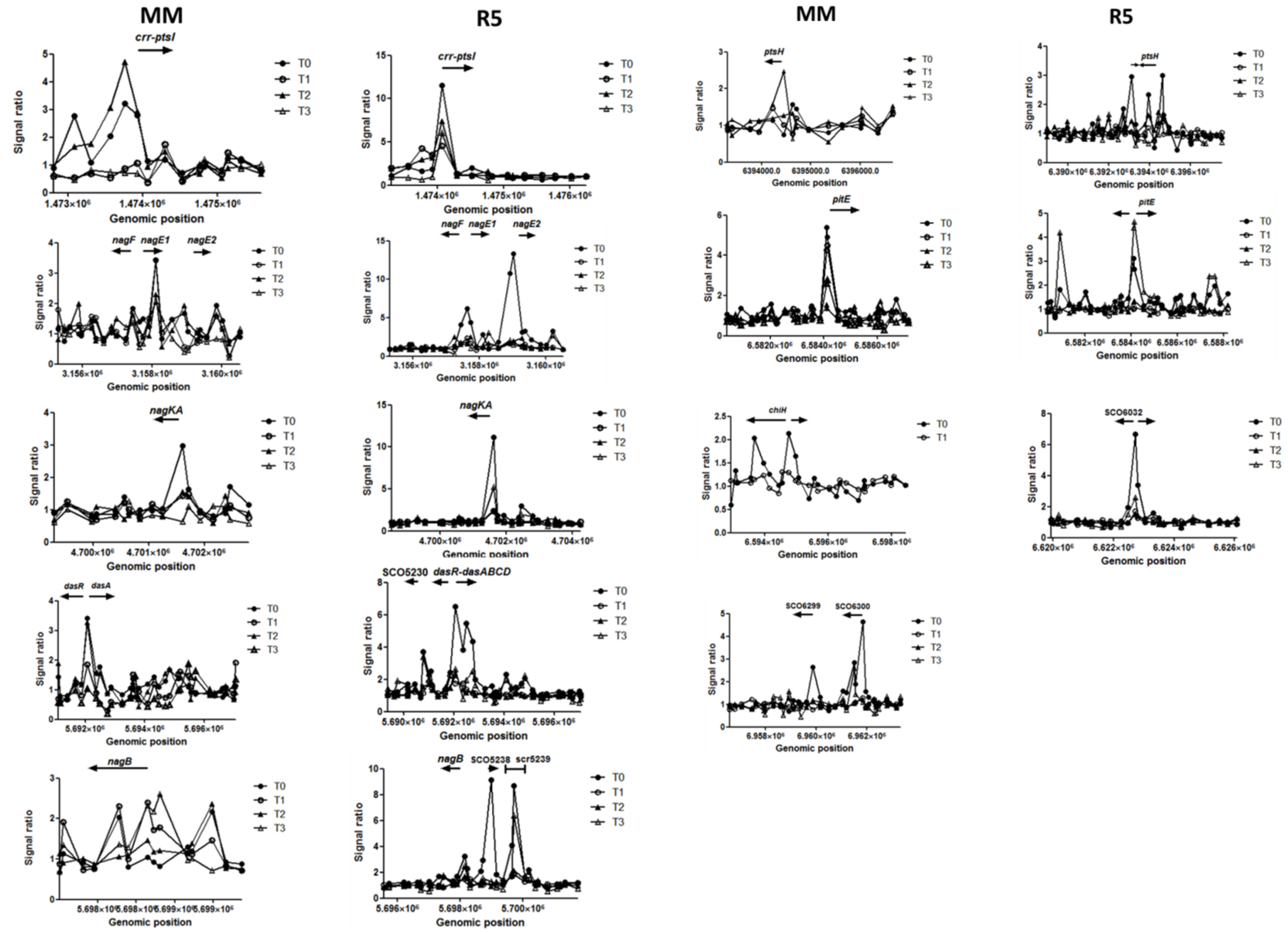

Supplement: S3 Fig — (PDF) [file pone.0122479.s003.pdf]
