# Supplementary material for: Genome-Wide Analysis of In Vivo Binding of the Master Regulator DasR in Streptomyces coelicolor Identifies Novel Non-Canonical Targets
Source: PLoS One. 2015 Apr 15;10(4):e0122479. doi: 10.1371/journal.pone.0122479 (PMC4398421; doi:10.1371/journal.pone.0122479)

S4 Fig. Chip-on-chip data for selected targets to which DasR remained bound in response to addition of GlcNAc. Samples were collected prior to (T_0, closed circles) and 30 (T_1, closed squares), 60 (T_2, closed triangle) or 120 min (T_3, closed inverted triangle) after addition of GlcNAc. Constant binding of DasR is shown to the promoter regions of *pyrR-bldD*, the upstream regions of the tRNA genes/operons tRNAGlu/Asp/Phe, tRNAGln/Glu and tRNAMet, and *secE* for the preprotein translocase subunit SecE. Note that binding to the *pyrR-bldD* intergenic region was enhanced.

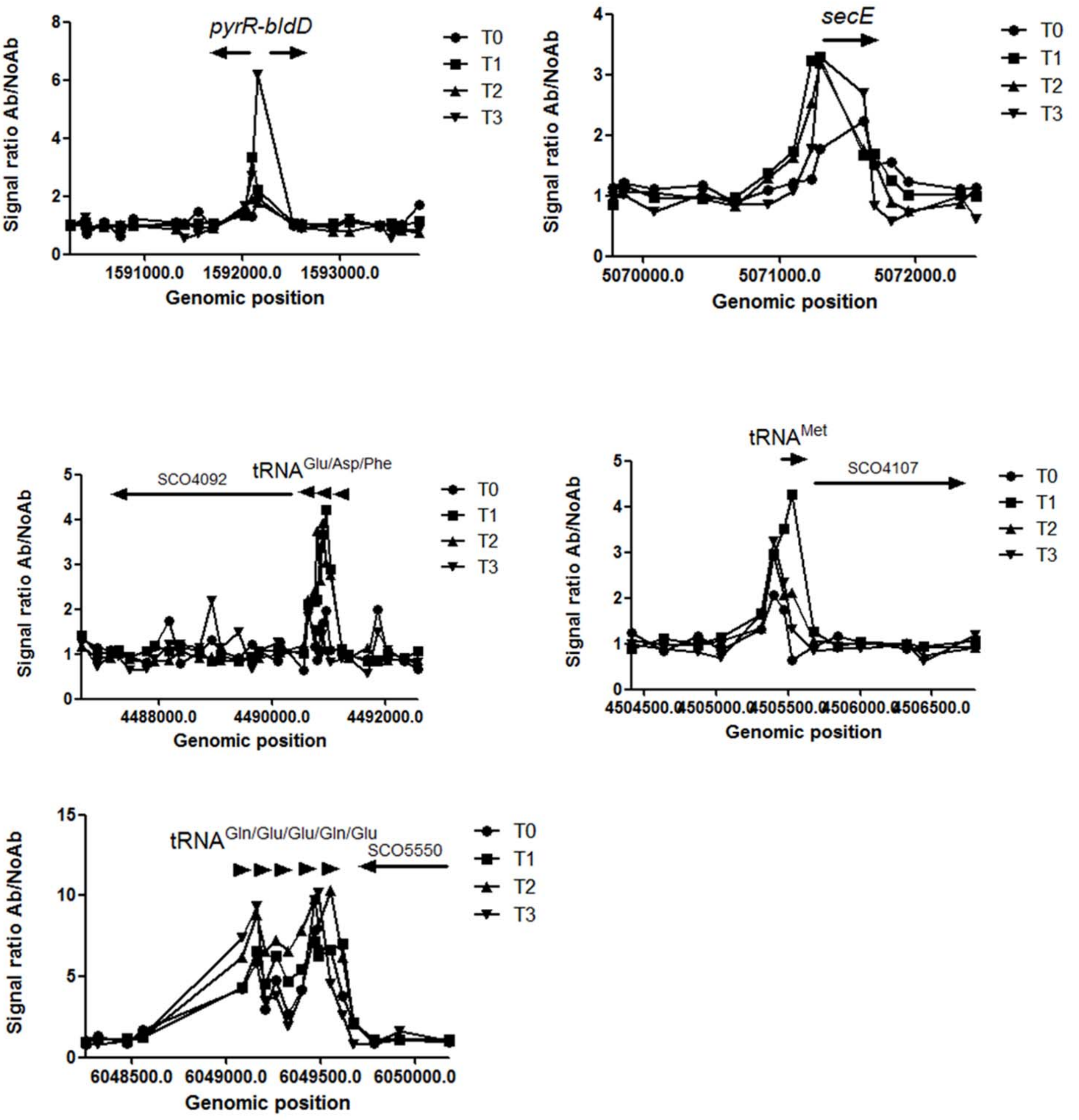

Supplement: S4 Fig — (PDF) [file pone.0122479.s004.pdf]
