# Supplementary material for: Genome-Wide Analysis of In Vivo Binding of the Master Regulator DasR in Streptomyces coelicolor Identifies Novel Non-Canonical Targets
Source: PLoS One. 2015 Apr 15;10(4):e0122479. doi: 10.1371/journal.pone.0122479 (PMC4398421; doi:10.1371/journal.pone.0122479)

S5 Fig. Chip-on-chip data for selected targets for which DasR binding was induced in response to GlcNAc addition. Samples were collected prior to (T_0, closed circles) and 30 (T_1, closed squares), 60 (T_2, closed triangle) or 120 min (T_3, closed inverted triangle) after addition of GlcNAc. Enhanced binding of DasR to the upstream regions of: *cydA* for cytochrome oxidase subunit I; the cell division-related gene SCO2078; *dnaZ* for DNA polymerase II subunit gamma; and the gene for ncRNA scr3092./

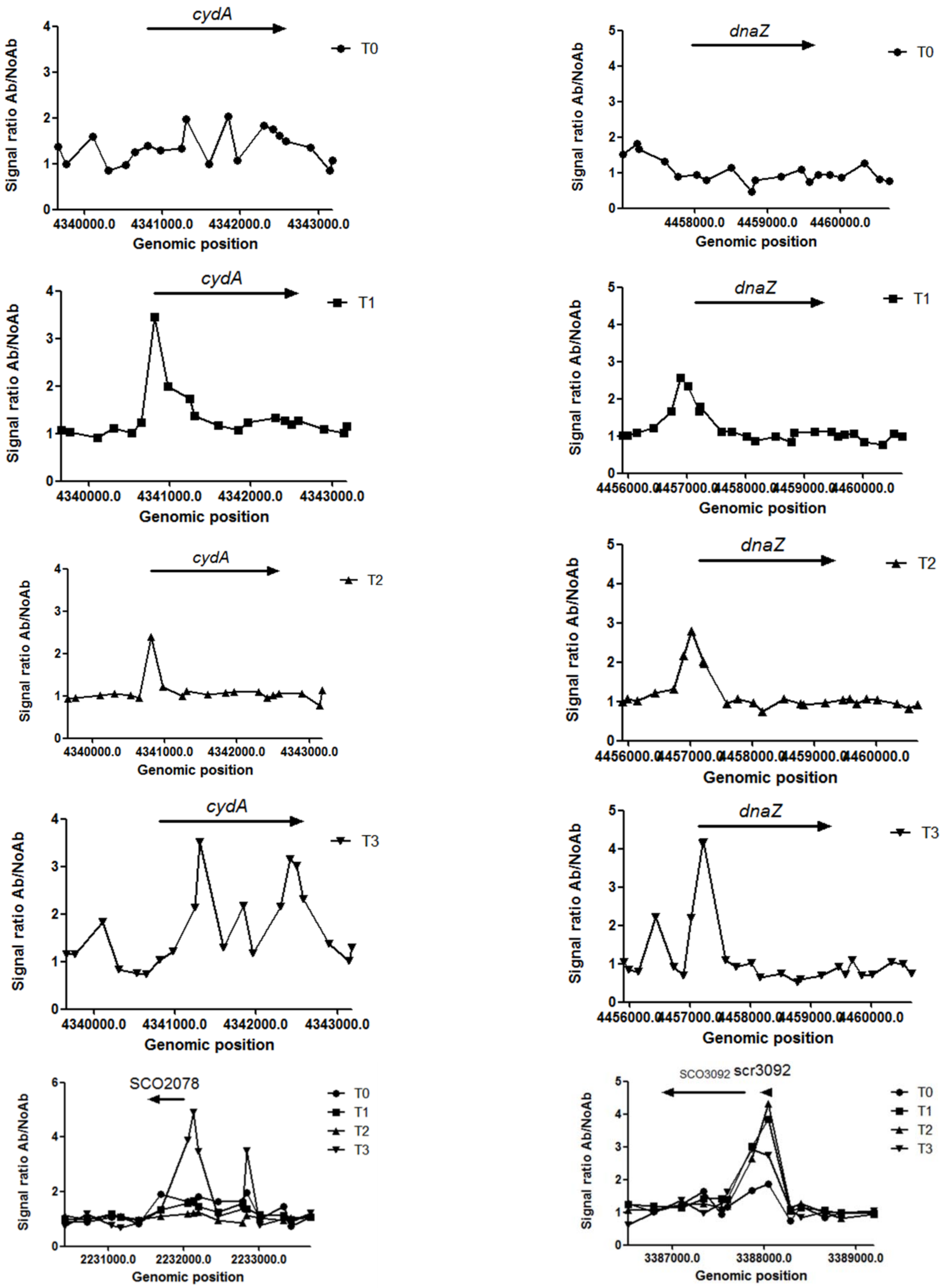

Supplement: S5 Fig — (PDF) [file pone.0122479.s005.pdf]
