# Supplementary material for: Genome-Wide Analysis of In Vivo Binding of the Master Regulator DasR in Streptomyces coelicolor Identifies Novel Non-Canonical Targets
Source: PLoS One. 2015 Apr 15;10(4):e0122479. doi: 10.1371/journal.pone.0122479 (PMC4398421; doi:10.1371/journal.pone.0122479)
Supplement: S1 Table — Expression, trend in microarray analysis. (PDF) [file pone.0122479.s006.pdf]

S1 Table. Binding by DasR as identified by genome-wide ChIP-on-chip experiments. Expression, trend in microarray analysis.
NB, no binding; B, binding; numbers between brackets indicate the number of probes that were signficantly enriched.

Nr	Gene ID	Annotation	Name	24h	54h	Expression
1	SCO0003 intragenic	putative DNA-binding protein		NB	B (1)	
2	SCO0005 intragenic	putative transposase		NB	B (1)	
3	SCO0018 upstream	hypothetical protein		NB	B (1)	
4	SCO0020 intragenic	putative transposase		NB	B (2)	
5	SCO0046 intragenic	putative hydrolase		NB	B(1)	
6	SCO0050 intragenic	hypothetical protein		NB	B(1)	down 54h
7	SCO0053 upstream	putative transposase		NB	B(1)	
8	SCO0055 intragenic	putative membrane-associated oxidoreductase		NB	B(1)	
9	SCO0072 intragenic	putative secreted protein		NB	B(1)	
10	SCO0073 upstream	hypothetical protein		NB	B(1)	
11	SCO0079 intragenic	putative integral membrane transport protein		NB	B(1)	down 54h
12	SCO0086 upstream	putative ribosylglycohydrolase (putative secreted protein)		NB	B(1)	
13	SCO0090 intragenic	putative transposase		NB	B(1)	up 42h
14	SCO0096 upstream	putative noncomposite transposon transposase		NB	B(1)	down 54h
15	SCO0097 upstream	putative integral membrane protein		NB	B(3)	
16	SCO0103 intragenic	probable flavohemoprotein		NB	B(1)	
17	SCO0130 intragenic	possible beta-lactamase		NB	B(1)	
18	SCO0166 intragenic	possible regulator		NB	B(1)	down 36h
19	SCO0239 intragenic	hypothetical protein		NB	B(1)	down 54h
20	SCO0261 upstream	probable acetyltransferase (SCO0261)		NB	B(2)	
21	SCO0271 intragenic	possible binding-protein-dependent transport protein		NB	B(1)	
22	SCO0287 intragenic	possible regulatory protein		NB	B(1)	
23	SCO0311 intragenic	probable ligase		NB	B(1)	
24	SCO0319 intragenic	hypothetical protein		NB	B(1)	
25	SCO0368 intragenic	transposase		NB	B(1)	
26	SCO0382 upstream	probable UDP-glucose/GDP-mannose family dehydrogenase (putative secreted protein)		B(1)	NB	up 30-42h
27	SCO0432 upstream	probable secreted peptidase		B(1)	NB	
28	SCO0511 intragenic	possible dehydrogenase (putative secreted protein)		B(1)	NB	
29	SCO0588 intragenic	possible sensor kinase	*cvnA11*	B(1)	NB	
30	SCO0615 intragenic	putative secreted protein		B(1)	NB	
31	SCO0661 intragenic	possible binding protein dependent transport protein		NB	B(1)	
32	SCO0915 upstream	hypothetical protein		NB	B(1)	
33	SCO0923 intragenic	probable reductase flavoprotein subunit		NB	B(1)	
34	SCO0947 intragenic	possible integral membrane protein		NB	B(1)	
35	SCO0961 intragenic	glucose-1-phosphate adenylyltransferase	*glgC*	NB	B(1)	
36	SCO0968 upstream	hypothetical protein		NB	B(1)	

37	SCO0976 intragenic	hypothetical protein		B(1)	NB	
38	SCO1061 intragenic	probable bifunctional protein (secreted sugar binding protein/sugar hydrolase)		NB	B(1)	
39	SCO1109 intragenic	probable oxidoreductase		NB	B(1)	
40	SCO1116 upstream	hypothetical protein (SCO1116)		NB	B(1)	
41	SCO1166-67 intragenic	putative integral membrane protein (SCO1166)<br>probable helicase (fragment) (SCO1167)		NB	B(1)	
42	SCO1276 upstream	RNA polymerase ECF sigma factor	*sigJ*	NB	B(2)	down 54h
43	SCO1321 upstream	elongation factor TU-3	*tuf3*	B(1)	NB	up 24h
44	SCO1327 intragenic	possible integral membrane protein		NB	B(1)	
45	SCO1390-SCO1391 upstream	PTS sugar phosphotransferase component IIA<br>phosphoenolpyruvate-protein phosphotransferase PtsI	*crr*<br>*ptsI*	B(1)<br>B(1)	B(1)<br>NB	
46	SCO1429 upstream	chitinase	*chiD*	B(2)	B(1)	
47	SCO1444 upstream	chitinase	*chiI*	B(1)	B(1)	
48	SCO1471 intragenic	putative transposase		NB	B(1)	
49	SCO1488-89 intergenic	pyrimidine operon regulatory protein<br>DNA-binding protein BldD	*pyrR*<br>*bldD*	NB	B(1)	
50	SCO1499 intragenic	possible integral membrane protein		NB	B(1)	
51	SCO1538 intragenic	probable transport system membrane protein		NB	B(1)	
52	SCO1645 intragenic	conserved hypothetical protein		NB	B(2)	
53	SCO1793 intragenic	putative secreted protein		NB	B(1)	down 54h
54	SCO2013 upstream	probable two-component system response regulator		NB	B(1)	
55	SCO2062-63 intragenic	hypothetical protein (SCO2062)<br>putative small hydrophilic protein (SCO2063)		NB	B(1)	up 54h
56	SCO2064-65 intragenic	DNA polymerase III alpha chain (SCO2064)<br>conserved hypothetical protein	*dnaE*	NB	B(1)	
57	SCO2078 upstream+intragenic	putative membrane protein		NB	B(2)	up 24 and 54h
58	SCO2107 intragenic	possible oxidoreductase		NB	B(1)	
59	SCO2125 upstream	hypothetical protein		NB	B(1)	
60	SCO2188 intragenic	possible peptidase (putative secreted protein)		B(1)	NB	
61	SCO2198 intragenic	glutamine synthetase I	*glnA*	NB	B(1)	up 24h
62	SCO2371 intragenic	pyruvate dehydrogenase E1 component	*aceE2*	NB	B(1)	
63	SCO2381 upstream+intragenic	conserved hypothetical protein		NB	B	
64	SCO2474 intragenic	putative metalloproteinase (putative secreted protein)		B(1)	NB	
65	SCO2490 intragenic	putative dehydrogenase (putative secreted protein)		B(1)	NB	
66	SCO2494-95 intragenic	putative pyruvate phosphate dikinase (SCO2494)<br>putative membrane protein (SCO2495)		NB	B(1)	
67	SCO2503 upstream	chitinase	*chiJ*	B(2)	B(1)	

68	SCO2504-05 intergenic	glycyl-tRNA synthetase<br><br>putative ABC-transporter metal-binding lipoprotein (SCO2505)	*glyS*	NB	B(2)	
69	SCO2528-29 intragenic	2-ispoprylmalate synthase (SCO2528)<br>putative metalloprotease (SCO2529)	*leuA*	NB	B(1)	down 24h
70	SCO2532 upstream+intragenic	PhoH-like protein		NB	B(2)	up 24h
71	SCO2561 upstream	possible long-chain fatty-acid CoA ligase		NB	B(1)	
72	SCO2562-63 intergenic	GTP-binding protein<br>30s ribosomal protein S20 (SCO2563)	*lepA*<br>*rpsT*	NB	B(1)	
73	SCO2603 intragenic	putative integrase; Contains TTA leucine codon, possible target for bldA regulation		NB	B(1)	
74	SCO2629 intragenic	putative membrane protein		B(1)	NB	
75	SCO2632 intragenic	putative transposase		NB	B(1)	
76	SCO2650 upstream	putative secreted protein		NB	B(1)	
77	SCO2669 intragenic	hypothetical protein		B(1)	NB	
78	SCO2681 intragenic	possible ATP/GTP-binding protein		NB	B(1)	
79	SCO2724-25 intergenic	putative amino acid transporter (SCO2724)<br>possible lipoprotein		NB	B(1)	
80	SCO2729 intragenic	possible acetyltransferase		B(1)	NB	down 54h
81	SCO2738 intragenic	hypothetical protein		NB	B(1)	
82	SCO2783 intragenic	probable monooxygenase	*desB,desC*	NB	B(1)	
83	SCO2833 upstream	secreted chitin binding protein	*chb*	B(1)	B(1)	
84	SCO2836 intragenic	possible glycosyl transferase		NB	B(1)	
85	SCO2868 intragenic	conserved hypothetical protein		NB	B(1)	
86	SCO2898 upstream	molecular chaperone	*sugE*	B(1)	B(1)	
87	SCO2902 intragenic	conserved hypothetical protein		B(1)	NB	
88	SCO2904 upstream	putative ribonuclease PH	*rph*	B(1)	NB	
89	SCO2906 upstream	PTS transmembrane component NagE1	*nagE1*	B(2)	B(2)	up 42h
90	SCO2907 upstream	PTS transmembrane component NagE2	*nagE2*	B(2)	B(2)	up 24-54h
91	SCO2945 intragenic	putative sugar transporter membrane protein		NB	B(1)	
92	SCO2946 upstream	putative sugar transporter sugar-binding protein		NB	B(1)	
93	SCO2977 intragenic	hypothetical protein		NB	B(2)	up 30h
94	SCO2986-87 upstream	conserved hypothetical protein<br>putative regulatory protein (SCO2987)	*ohrA*<br>*ohrR*	NB	B(1)	
95	SCO2990-91 intragenic	hypothetical protein (SCO2990)<br>hypothetical protein (SCO2991)		B(1)	NB	
96	SCO3005 intragenic	preprotein translocase	*secA*	NB	B(1)	

97	SCO3043 upstream	conserved hypothetical protein		NB	B(1)	
98	SCO3067-68 intergenic	putative anti anti sigma factor<br>putative RNA polymerase sigma factor	*arsI*<br>*sig15,sigI*	NB	B(1)	
99	SCO3079 upstream	putative thiolase		B(1)	NB	down 30h
100	SCO3092 upstream	ncRNA		B(3)	B(4)	up 36-54h
101	SCO3112 intragenic	unknown		NB	B(1)	up 42-54h
102	SCO3138-39 intergenic	galactose-1-phosphate uridylyltransferase (SCO3138)<br>putative sodium:solute symporter (SCO3139)	*galT*	NB	B(1)	down 42-54h
103	SCO3218 downstream	putative small conserved hypothetical protein		B(1)	NB	down 24h;54h
104	SCO3230 intragenic	CDA peptide synthetase I	*cdaPSI*	NB	B(3)	
105	SCO3231 intragenic	CDA peptide synthetase II	*cdaPSII*	NB	B(1)	
106	SCO3261 intragenic	putative ATP-binding protein		NB	B(1)	down 24h
107	SCO3262 intragenic	hypothetical protein		NB	B(3)	down 24h
108	SCO3266 intragenic	hypothetical protein		NB	B(1)	up (30h-54h)
109	SCO3305 intragenic	putative membrane protein		NB	B(1)	
110	SCO3323 upstream	putative RNA polymerase sigma factor	*bldN*	NB	B(1)	up 42-54h
111	SCO3365 upstream	unknown		NB	B(1)	
112	SCO3398 upstream	probable dihydropteroate synthase	*folP*	B(1)	B(1)	
113	SCO3468 intragenic	transposase		NB	B(1)	
114	SCO3469 intragenic	transposase		NB	B(3)	
115	SCO3471 intragenic	extracellular agarase precursor	*dagA*	NB	B(1)	
116	SCO3473 intragenic	probable aldolase		NB	B(1)	down 42h
117	SCO3476 intragenic	probable dehydrogenase		NB	B(1)	down 36-54h
118	SCO3479 upstream	possible beta-galatosidase		B(1)	NB	down 36-54h
119	SCO3482 intragenic	possible sugar-permease (SCO3482)		NB	B(2)	down 30-54h
120	SCO3484 intragenic	possible secreted sugar-binding protein,		NB	B(1)	down 30-54h
121	SCO3487 upstream	putative hydrolase		NB	B(1)	down 30-54h
122	SCO3490 intragenic	transposase		NB	B(2)	
123	SCO3498 intragenic	putative secreted protein		NB	B(1)	
124	SCO3503 intragenic	putative binding protein dependent transport protein		NB	B(1)	
125	SCO3504 intragenic	putative binding protein dependent transport protein		NB	B(1)	down 54h
126	SCO3510-11 intergenic	putative DNA methylase (SCO3510)<br>possible lipoprotein (SCO3511)		NB	B(1)	
127	SCO3545 upstream	putative transferase		B(1)	NB	
128	SCO3567 intragenic	probable serine protease		NB	B(1)	
129	SCO3649 upstream	putative fructose 1,6-bisphosphate aldolase	*fba*	B(1)	NB	up 54h
130	SCO3664 upstream	possible regulatory protein		NB	B(2)	
131	SCO3679 upstream	regulatory protein		B(2)	B(1)	
132	SCO3713 upstream	hypothetical protein		NB	B(1)	
133	SCO3718 intragenic	probable cation transport system component	*kdpA*	NB	B(1)	
134	SCO3746 intragenic	putative membrane protein		NB	B(1)	
135	SCO3787 upstream	putative integral membrane protein		B(1)	NB	

136	SCO3888 intragenic	conserved hypothetical protein		NB	B(3)	
137	SCO3922 intragenic	putative membrane protein		NB	B(2)	
138	SCO3938 intragenic	probable thiamin biosynthesis protein ThiC	*thisC*	B(1)	NB	down 30h
139	SCO3986 intragenic	putative GntR-family transcriptional regulator		NB	B(2)	up 24-54h
140	SCO3989 intragenic	possible ribonuclease inhibitor		NB	B(1)	up 24-54h
141	SCO3993 intragenic	hypothetical protein		NB	B(1)	up 30h,42h
142	SCO4089-90 intergenic	valine dehydrogenase (SCO4089)<br>conserved hypothetical protein (SCO4090)	*vdh*	NB	B(1)	
143	SCO4138 intragenic	phosphate transport protein	*pitH*	NB	B(1)	
144	SCO4152 intragenic	possible secreted 5'-nucleotidase		NB	B(1)	
145	SCO4183 intragenic	putative transposase		B(1)	B(1)	
146	SCO4219 upstream	putative hydrolase		NB	B(2)	
147	SCO4231-32 intergenic	putative lipoprotein (SCO4231)<br>putative transcriptional factor regulator (SCO4232)		NB	B(1)	
148	SCO4281 intragenic	conserved hypothetical protein		NB	B(1)	
149	SCO4284-85	N-acetylglucosamine-6-phosphate deacetylase (SCO4284)	*nagA*	B(3)	B(2)	
		N-acetylglucosamine kinase	*nagK*			up 24-54h
150	SCO4333-34 intergenic	putative integral membrane protein (SCO4333)<br>possible integral membrane protein (SCO4334)		NB	B(1)	
151	SCO4342 intragenic	conserved hypothetical protein		NB	B(1)	down 54h
152	SCO4344 upstream + intragenic	transposase		NB	B(2)	
153	SCO4354 intragenic	hypothetical protein		NB	B(1)	
154	SCO4391-92 intragenic	conserved hypothetical protein (SCO4391)		NB	B(1)	
155	SCO4432 intragenic	hypothetical protein		NB	B(1)	
156	SCO4466 intragenic	hypothetical protein		NB	B(1)	
157	SCO4521 intragenic	putative secreted protein		NB	B(2)	
158	SCO4526 intragenic	possible protein kinase		NB	B(1)	
159	SCO4529 intragenic	putative integral membrane protein		NB	B(1)	
160	SCO4540 upstream	hypothetical protein		NB	B(3)	down 54h
161	SCO4589 upstream	probable aminopeptidase (putative secreted protein)		B(1)	NB	
162	SCO4630 intragenic	hypothetical protein		NB	B(1)	up 24h-42h
163	SCO4632 intragenic	putative ATP/GTP binding protein		NB	B(1)	up42-54h
164	SCO4644 downstream	SCO4644-add, adenosine deaminase;	*add*	NB	B(1)	
165	SCO4698 intragenic	putative insertion element IS1652 transposase		NB	B(2)	
166	SCO4699 intragenic	putative Rhs protein		NB	B(3)	up 30h
167	SCO4800 intragenic	isobutyryl CoA mutase	*icm*	NB	B(1)	up 30-54h
168	SCO4816 intragenic	hypothetical protein (fragment)		NB	B(1)	
169	SCO4859 intragenic	hypothetical protein		NB	B(1)	
170	SCO4874 intragenic	putative integral membrane protein		NB	B(1)	
171	SCO4914 intragenic	putative deoxyribose-phosphate aldolase		NB	B(1)	
172	SCO4915 upstream	putative integral membrane protein		NB	B(1)	

173	SCO4934 upstream	possible lipoprotein		NB	B(1)	up 24;36h
174	SCO4979 upstream	probable phosphoenolpyruvate carboxykinase		NB	B(1)	
175	SCO4987-88 intergenic	putative D-amino acid deaminase (SCO4987)		NB	B(1)	
		putative carbohydrate kinase		NB	B(1)	
176	SCO4997 intragenic	hypothetical protein				down 54h
177	SCO5003-04 intergenic	chitinase (SCO5003)	*chiA*	B(3)	B(1)	
		hypothetical protein (SCO5004)				
178	SCO5013 intragenic	putative secreted protein		NB	B(1)	
179	SCO5022 intragenic	putative lipoprotein		NB	B(1)	
180	SCO5032 intragenic	alkyl hydroperoxide reductase	*ahpC*	NB	B(1)	
181	SCO5117 intragenic	possible peptide transport system secreted peptide-binding protein		NB	B(1)	down 36h
182	SCO5161 upstream	conserved hypothetical protein		B(1)	B(1)	
183	SCO5189 intragenic	hypothetical protein		NB	B(1)	up 24-30h
184	SCO5231-32 intergenic	DasR	*dasR*	B(5)	B(4)	
		sugar binding protein DasA	*dasA*			up 24-54h
185	SCO5236 upstream	glucosamine phosphate isomerase	*nagB*	B(2)	NB	up 24-54h
186	SCO5237-38 intergenic	putative oxidoreductase (SCO5237)		B(2)	NB	
		putative TetR-family protein (SCO5238)				
187	SCO5239 upstream	scr5239		B(3)	B(2)	
188	SCO5300 intragenic	conserved hypothetical protein		B(1)	B(1)	
189	SCO5322 intragenic	conserved hypothetical protein;		B(1)	NB	
190	SCO5331 intragenic	possible DNA methylase		NB	B(1)	
191	SCO5332 intragenic	hypothetical protein		NB	B(1)	up 30h
192	SCO5342 intragenic	spdB2 protein		NB	B(1)	
193	SCO5365 intragenic	putative transferase		NB	B(1)	
194	SCO5368 upstream	ATP synthase C chain	*artpE*	NB	B(1)	up 24;54h
195	SCO5376 upstream	chitinase	*chiC*	B(3)	NB	
196	SCO5411 intragenic	putative integrase/recombinase;		NB	B(1)	
197	SCO5428 intragenic	putative integral membrane transport protein		NB	B(1)	
198	SCO5431 intragenic	putative secreted nucleosidase		B(1)	NB	
199	SCO5443 intragenic	possible alpha-amylase	*pep1A*	NB	B(2)	
200	SCO5458 intragenic	putative lipoprotein		NB	B(1)	
201	SCO5535 upstream	probable carboxyl transferase	*accB*	B(1)	NB	up 54h
202	SCO5550 downstream	tRNA-Gln-Glu		B(8)	B(9)	
203	SCO5564 intragenic	rpmB, 50S ribosomal protein L28		NB	B(1)	down 54h
204	SCO5613 intragenic	hypothetical protein		NB	B(1)	
205	SCO5616 intragenic	hypothetical protein		NB	B(1)	down 54h
206	SCO5641 intragenic	transposase		NB	B(1)	

207	SCO5644 intragenic	hypothetical protein		NB	B(3)	down 24;54h
208	SCO5672 upstream	hypothetical protein		NB	B(1)	down 30-54h
209	SCO5673 upstream	chitinase	*chiB*	B(1)	B(1)	
210	SCO5702-03 intragenic	putative lipoprotein (SCO5702)<br>hypothetical protein (SCO5703)		NB	B(1)	
211	SCO5727 intragenic	hypothetical protein		NB	B(1)	
212	SCO5728 intragenic	putative secreted protein		NB	B(1)	
213	SCO5751 upstream	putative membrane protein		NB	B(2)	
214	SCO5827 intragenic	probable transmembrane transport protein		NB	B(1)	
215	SCO5840 upstream	probable transcriptional regulator		B(1)	NB	
216	SCO5841-42 intragenic	phosphocarrier protein hpr (SCO5841)<br><br>conserved hypothetical protein (SCO5842)	*ptsH*	B(4)	B(3)	up 24h-54h
217	SCO5874 intragenic	putative membrane protein		NB	B(1)	
218	SCO5916 intragenic	hypothetical protein		NB	B(1)	
219	SCO5922 upstream	possible oxidoreductase		NB	B(1)	
220	SCO5952 intragenic	putative membrane protein		NB	B(1)	
221	SCO6004-05 intergenic	secreted ATP/GTP binding protein (SCO6004)<br>lipoprotein (SCO6005)	 *ngcE, pitE*	NB	B(1)	 up 24-54h
222	SCO6012-13 intergenic	chitinase<br><br>probable 1-deoxyxylulose-5-phosphate synthase	*chiH*	B(3)	NB	
223	SCO6032-33 intergenic	putative hydrolase (SCO6032)<br>hypothetical protein with phytanoyl-CoA dioxygenase domain (SCO6033)		B(2)	B(2)	
224	SCO6127 intragenic	putative carboxylesterase		B(1)	B(1)	
225	SCO6167 intragenic	proline rich protein (putative membrane protein)		B(1)	NB	
226	SCO6169-70 intergenic	putative regulatory protein (SCO6169)<br>probable oxidoreductase subunit (SCO6170)		NB	B(1)	
227	SCO6236 intragenic	possible DNA-binding protein		NB	B(1)	
228	SCO6240 intragenic	conserved hypothetical protein		NB	B(2)	up 24-30h;42h
229	SCO6273 intragenic	type I polyketide synthase	*cpkC*	NB	B(2)	
230	SCO6274 coding	type I polyketide synthase	*cpkB*	NB	B(7)	
231	SCO6275 intragenic	type I polyketide synthase (fragment)	*cpkA*	NB	B(1)	
232	SCO6300 upstream	secreted ß-N-acetylglucosaminidase		B(3)	NB	
233	SCO6304 intragenic	putative oxidoreductase		NB	B(1)	
234	SCO6321 intragenic	possible integral membrane protein		NB	B(1)	
235	SCO6325 intragenic	putative membrane protein		NB	B(1)	
236	SCO6336 intragenic	hypothetical protein		NB	B(1)	
237	SCO6344-45 intergenic	putative secreted amidase (SCO6344)		B(4)	NB	

		putative secreted chitinase (SCO6345)	*chi*			
238	SCO6373 intragenic	putative integral membrane protein		NB	B(2)	down 36h
239	SCO6377 upstream	putative lipoprotein		NB	B(1)	down 36h
240	SCO6381 intragenic	putative lipoprotein		NB	B(1)	
241	SCO6383 intragenic	putative integral membrane protein		NB	B(1)	
242	SCO6384 intragenic	putative integral membrane lysyl-tRNA synthetase		NB	B(1)	down 24h
243	SCO6385 intragenic	probable integral membrane protein,		NB	B(1)	
244	SCO6387 intragenic	hypothetical protein		NB	B(1)	
245	SCO6390 intragenic	putative integral membrane protein		NB	B(1)	down 36h
246	SCO6393 intragenic	putative transposase		NB	B(1)	up 42h
247	SCO6400 intragenic	putative IS117 transposase		NB	B(2)	
248	SCO6401 intragenic	conserved hypothetical protein		NB	B(1)	down 54h
249	SCO6470 intragenic	hypothetical protein		NB	B(1)	
250	SCO6474 upstream	possible transcriptional regulator;		B(1)	NB	
251	SCO6486 intragenic	putative transport associated protein		B(1)	NB	
252	SCO6512 intragenic	ABC transporter ATP-binding protein		B(1)	NB	
253	SCO6513 intragenic	hypothetical protein		B(1)	NB	
254	SCO6622 intragenic	putative ATP/GTP binding protein		NB	B(1)	
255	SCO6625 intragenic	hypothetical protein		NB	B(1)	
256	SCO6627 intragenic	putative adenine-specific DNA methyltransferase gene	*pglX*	NB	B(4)	
257	SCO6632 upstream	hypothetical protein		NB	B(1)	
258	SCO6640 intragenic	putative ATP-dependent helicase		NB	B(1)	
259	SCO6807 upstream	hypothetical protein		NB	B(1)	
260	SCO6834 intragenic	possible thioredoxin reductase	*trxB2*	NB	B(1)	
261	SCO6859 intragenic	hypothetical protein		NB	B(1)	
262	SCO6860 intragenic	hypothetical protein		NB	B(1)	
263	SCO6861 intragenic	protein kinase-like protein	*pk3*	NB	B(2)	
264	SCO6862 intragenic	hypothetical protein		NB	B(1)	up 42h
265	SCO6871 intragenic	putative lipoprotein		NB	B(1)	
266	SCO6876 intragenic	hypothetical protein		NB	B(1)	
267	SCO6908 intragenic	hypothetical protein		NB	B(1)	up 30;42h
268	SCO6948 intragenic	hypothetical protein		NB	B(1)	up 42h
269	SCO6952 intragenic	hypothetical protein		NB	B(3)	up 24-30h
270	SCO6953 intragenic	conserved hypothetical protein		NB	B(2)	
271	SCO7018 intragenic	hypothetical protein		NB	B(2)	down 54h
272	SCO7019 intragenic	secreted alpha-amylase (EC 3.2.1.1)	*amlI*	NB	B(1)	
273	SCO7037 intragenic	putative secreted protein;		NB	B(1)	
274	SCO7056 upstream	possible gntR-family transcriptional regulator		B(2)	B(2)	
275	SCO7059 upstream	putative oxidoreductase		B(1)	NB	
276	SCO7077 intragenic	putative integral membrane protein		NB	B(1)	

277	SCO7080 intragenic	putative insertion element transposase		NB	B(1)	
278	SCO7120 intragenic	ubiquinol-cytochrome C reductase cytochrome B subunit	*qcrB2*	NB	B(1)	
279	SCO7165 intragenic	probable sugar-binding integral membrane transport protein		NB	B(1)	
280	SCO7197 intragenic	probable amino acid transport integral membrane protein		NB	B(1)	
281	SCO7225 upstream	chitinase	*chi*	B(2)	B(1)	
282	SCO7226 upstream	putative integral membrane protein		B(1)	B(1)	
283	SCO7227 intragenic	possible secreted protein		B(1)	NB	
284	SCO7263 upstream	chitinase	*chiF*	B(1)	NB	
285	SCO7274 upstream	possible membrane protein		B(1)	NB	
286	SCO7279 intragenic	possible DNA-binding protein	*popR*	B(1)	NB	
287	SCO7330 intragenic	putative membrane protein		B(1)	NB	up 42h
288	SCO7335 intragenic	possible alpha-amylase	*pep1B*	NB	B(2)	down 36h
289	SCO7350 intragenic	putative membrane efflux protein		NB	B(1)	
290	SCO7392 intragenic	hypothetical protein		NB	B(1)	
291	SCO7410 intragenic	putative binding-protein dependent transport protein.		B(1)	NB	
292	SCO7416 upstream	hypothetical protein		B(1)	NB	
293	SCO7493 intragenic	conserved hypothetical protein		B(1)	NB	
294	SCO7494-95 itergenic	putative membrane protein (SCO7494)<br>pseudogene, hypothetical protein		NB	B(1)	
295	SCO7520 upstream	putative integral membrane protein		B(1)	NB	
296	SCO7742 intragenic	putative MarR-family transcriptional regulator		NB	B(1)	
297	SCO7743 intragenic	hypothetical protein		NB	B(1)	down 54h
298	SCO7752 intragenic	putative integral membrane protein		NB	B(2)	
299	SCO7775 intragenic	putative secreted protein		NB	B(2)	
300	SCO7785 intragenic	putative transcriptional regulator		NB	B(1)	
301	SCO7792 intragenic	hypothetical protein		B(1)	NB	up 42h;down 54h
302	SCO7798 intragenic	transposase		NB	B(2)	down 54h
303	SCO7803 intragenic	putative insertion element transposase		NB	B(1)	
304	SCO7804 upstream	putative membrane protein		NB	B(1)	
305	SCO7826 upstream	hypothetical protein		NB	B(1)	down 54h
306	SCO7827 intragenic	putative transposase		NB	B(1)	
307	SCO7837-38	putative membrane protein (SCO7837)<br>conserved hypothetical protein (SCO7838)		NB	B(1)	down 54h
308	SCO7842 intragenic	putative transposase		NB	B(1)	
309	SCO7844 intragenic	putative DNA-binding protein		NB	B(1)	
310	SCO4123[d]	rrnA		-	B (>2)	
311	SCO1792[d]	rrnB		-	B (>2)	
312	SCO1390	rrnC		-	B (>2)	

313	SCO5746	rrnE		-	B (>2)	
314	SCO3334	rrnF		-	B (>2)	
		**Total**		**21**	**56**	
