# Supplementary material for: Genome-Wide Analysis of In Vivo Binding of the Master Regulator DasR in Streptomyces coelicolor Identifies Novel Non-Canonical Targets
Source: PLoS One. 2015 Apr 15;10(4):e0122479. doi: 10.1371/journal.pone.0122479 (PMC4398421; doi:10.1371/journal.pone.0122479)
Supplement: S2 Table — (PDF) [file pone.0122479.s007.pdf]

S2 Table. DasR binding before and after addition of GlcNAc to liquid-grown MM and R5 cultures. T0, before induction; T1, T2, T3; time 30, 60 and 120 min after the addition of GlcNAc. B, binding; N, no binding; B_r. binding reduced; B_e, binding enhanced.

												Trend of DasR binding after addition of GlcNAc **#**	
**Nr**	**Gene ID**	**Name**	**Function**	**MM**				**R5**				**MM**	**R5**
				T0	T1	T2	T3	T0	T1	T2	T3		
			**PTS, GlcNAc metabolism, chitinases and sugar-related**										
1	SCO1390-SCO1391	*crr-ptsI*	PTS system sugar phosphotransferase component IIA	B	N	B	N	B	B	B	B	dissociation	2 fold reduction in binding
2	SCO1429	*chiD*	chitinase	N	N	N	N	B	N	N	B		dissociation
3	SCO1444	*chiI*	chitinase	N	N	N	N	N	N	N	N		
4	SCO2503	*chiJ*	chitinase	N	N	N	N	B	N	N	B		dissociation
5	SCO2833	*chb*	secreted chitin binding protein	B	N	B	N	B	N	B	N	dissociation	dissociation
6	SCO2906	nagE1	putative PTS transmembrane component	N	N	N	N	B	B	B	B		more than 2 fold reduction
7	SCO2907	nagE2	putative PTS transmembrane component	N	N	N	N	B	N	B	N		dissociation
8	SCO4284-sco4285	*nagAK*	putative GlcNAc-6P deacetylase (SCO4284); sugar kinase (SCO4285)	B	N	N	N	B	B	B	B	dissociation	more than 4 fold reduction
9	SCO5003-sco5004	*chiA*	chitinase (SCO5003);	B	N	N	N	B	N	N	N	dissociation	dissociation
			hypothetical protein (SCO5004)	B	N	N	N	B	N	N	N	dissociation	dissociation
10	SCO5231-SCO5232	*dasR*	putative gntR-family transcriptional regulator (SCO5231);	B	N	B	N	B	N	N	N	dissociation	dissociation
		dasA	putative sugar transporter sugar binding protein (SCO5232)										
11	SCO5236	*nagB*	putative glucosamine phosphate isomerase	N	N	N	N	B	N	N	B		dissociation
12	SCO5376	*chiC*	chitinase	N	N	N	N	N	N	N	N		
13	SCO5673	*chiB*	chitinase	B	B	B	B	B	N	N	N	constant binding	dissociation
14	SCO5841	*ptsH*	phosphocarrier protein hpr (SCO5841)	N	N	N	N	N	N	N	N		
15	SCO6004-SCO6005		putative secreted ATP/GTP binding protein (SCO6004);	B	B	B_r	B_r	B	N	N	B	two fold reduction at T2	dissociation
		ngcE, pitE	putative lipoprotein (SCO6005)										
16	SCO6012-SCO6013 intergenic	*chiH*	chitinase (SCO6012);	N	N	N	N	B	N	N	N		dissociation
			probable 1-deoxyxylulose-5-phosphate synthase (SCO6013)	N	N	N	N	B	N	N	N		
17	SCO6032-SCO6033 intergenic		putative hydrolase (SCO6032)	N	N	N	N	B	N	N	B		dissociation
			hypothetical protein with phytanoyl-CoA dioxygenase domain (SCO6033)										

18	SCO6300		secreted ß-N-acetylglucosaminidase	N	N	N	N	B	N	N	N		dissociation
19	SCO6344-SCO6345<br>intergenic	 <br>*chi*	putative secreted amidase (SCO6344)<br><br>putative secreted chitinase (SCO6345)	N	N	N	N	B	N	N	N		dissociation
20	SCO7225	*chi*	chitinase	N	N	N	N	B	N	N	N		dissociation
			**Development and antibiotic production**										
21	SCO3230	*cdaPSI*	CDA peptide synthetase I	B	N	B	N	B	N	N	B	dissociation	dissociation
22	SCO3231coding	*cdaPSII*	CDA peptide synthetase II	B	B	N	N	B	N	N	B	dissociation	dissociation
23	SCO6273	*cpkC*	probable type I polyketide synthase	B	B	N	B	N	N	N	N	binding induced at T1, but dissociaction at T2	
24	SCO6274	*cpkB*	probable type I polyketide synthase	B	B	N	B	N	N	N	N	binding induced at T1, but dissociaction at T3	
25	SCO5085	*actII-4*	actinorhodin cluster activator protein	B	N	B	N	B	N	N	B	dissociation	initial dissociation
26	SCO5877	*redD*	transcriptional regulator RedD	B	B_r	B_r	N	B	N	N	N	dissociation	dissociation
27	SCO5881coding	*redZ*	response regulator	B	N	B	N	B	N	N	B	dissociation	dissociation
28	SCO5114	*bldKC*	possible ABC transport system integral membrane protein	B	B_r	B_r	N	N	N	N	N	dissociation	
29	SCO4118-SCO4119<br>intergenic	*atrA*	possible tetR-family transcriptional regulator (SCO4118);<br>possible NADH dehydrogenase (SCO4119)	B	B	B_r	B_r	B	N	N	B	two fold reduction in bindir	dissociation
30	SCO2686-SCO2687<br>intergenic	 *ribA1*	possible luxR-family transcriptional regulator (SCO2686)<br>GTP cyclohydrolase II	B	B_r	B_r	N	B	N	N	B	dissociation	dissociation
31	SCO3258	*pra*	regulatory protein	B	B	B	B	N	N	N	N	constant binding	
			**Transposases and DNA recombination**										
32	SCO0098-SCO0099<br>intergenic		transposases	B	N	B	N	N	N	N	N	dissociation	
33	SCO0567		insertion element transposase	B	B	B	N	N	N	N	N	dissociation	
34	SCO1471		transposase	B	N	N	N	N	N	N	N	dissociation	
35	SCO1603		transposase	B	B_r	B_r	N	N	N	N	N	dissociation	
36	SCO3466		transposase	B	B	B	N	B	N	N	B	dissociation	dissociation
37	SCO3467		transposase	B	B	B	N	N	N	N	N	dissociation	
38	SCO3468		transposase	B	B	B	N	B	N	N	B	dissociation	dissociation
39	SCO6400		probable transposase	B	B_r	B_r	N	B	N	N	B	dissociation	initial dissociation
40	SCO6910-SCO6911<br>intergenic		transposases	B	B	B	N	B	N	N	B	dissociation	dissociation
			**Other targets**										
41	SCO1327		possible integral membrane protein	B	B	N	B	B	N	N	B	dissociation	dissociation
42	SCO1606		Penicillin Binding Protein (putative)	B	B	B_r	B_r	N	N	N	B	reduction in binding	binding
43	SCO1967-SCO1968<br>intergenic	 *glpQ2*	possible integral membrane protein (SCO1967)<br>probable secreted hydrolase (SCO1968)	B	B	B_r	B_r	B	N	N	B	reduction in binding	dissociation
44	SCO2078		putative membrane protein	B	B	B_r	B	N	N	N	B	reduction in binding	induction of binding
45	SCO2427-SCO2428<br>intergenic		probable secreted protein (SCO2427)<br>possible phosphate binding protein (SCO2428)	B	B	N	N	N	N	N	N	dissociation	

46	SCO2687		ribA1, GTP cyclohydrolase II	B	B_r	B_r	B_r	B	N	N	B	reduction in binding	dissociation
47	SCO2724-SCO2725 intergenic		possible amino acid transporter (SCO2724) putative lipoprotein (SCO2725)	B	B	B_r	B_r	N	N	N	N	reduction in binding	
48	SCO2741		possible secreted protein	B	B	N	N	N	N	N	N	dissociation	
49	SCO3249		probable acyl carrier protein	B	B	N	N	N	N	N	N	dissociation	
50	SCO3262		unknown	B	B	N	N	N	N	N	B	dissociation	binding
51	SCO3362		putative membrane protein	B	B	B	N	N	N	N	N	dissociation	
52	SCO3503		possible binding protein transport dependent protein	B	B	B_r	B_r	N	N	N	N	reduction in binding	
53	SCO3567		probable serine protease	B	B	B	N	N	N	N	B	dissociation	binding
54	SCO3594-SCO3595	*dldH* *ddlA2*	probable D-lactate dehydrogenase probable D-alanine:D-lactate ligase	B	B_r	B_r	N	B	N	N	B	dissociation	dissociation
55	SCO3748	*f40*	cold shock protein	B	B	B	N	B	N	N	B	dissociation	dissociation
56	SCO3871		possible decarboxylase	B	B	B	N	N	N	N	N	dissociation	
57	SCO4219		possible hydrolase (dienelactone hydrolase family)	B	B	B	B_r	N	N	N	N	reduction in binding	
58	SCO4540		unknown	B	B_r	B_r	B_r	B	N	N	B	reduction in binding	dissociation
59	SCO5014-SCO5015		possible secreted protein (SCO5014) putative secreted protein (SCO5015)	B	B	B	B_r	N	N	N	N	reduction in binding	
60	SCO5190	*wblC*	possible DNA-binding protein	B	B	B_r	B_r	N	N	N	N	reduction in binding	
61	SCO5423	*pyk2*	pyruvate kinase	B	B	B_r	B_r	N	N	N	N	two fold reduction in binding	
62	SCO6108	*fusH*	esterase	B	B	B_r	B_r	B	N	N	B	two fold reduction in bindin	dissociation
63	SCO6401		unknown	B	B	B_r	B_r	N	N	N	N	reduction in binding	
64	SCO6806		putative phage integrase	B	B	B_r	B_r	N	N	N	N		
65	SCO6816		possible ABC transporter binding lipoprotein	B	B	N	B	N	N	N	N	dissociation	
66	SCO7496-SCO7497 intergenic		conserved hypothetical integral membrane protein (SCO7496); possible transcriptional regulator (SCO7497)	B	B	N	N	N	N	N	N	dissociation	
67	SCO1020-SCO1021intergenic		hypothetical protein	N	N	N	N	B	N	N	B		dissociation
68	SCO3503		putative binding protein dependent transport protein (SCO3503)	B	B	B	N	B	N	B	B	dissociation	dissociation
69	SCO3660		hypothetical protein	B	B	B	B_r	B	N	N	B	reduction in binding	dissociation
70	SCO3844		putative secreted protein	B	B	B	N	B	N	N	B	dissociation	dissociation
71	SCO3978-SCO3979 intergenic		possible oxidoreductase (SCO3978) possible TetR-family transcriptional regulator (SCO3979)	N	N	N	N	B	N	N	B		dissociation
72	SCO3998-SCO3999 intergenic		unknown (SCO3998) possible lipoprotein (SCO3999)	B	B	N	B	B	N	N	B	dissociation	dissociation
73	SCO5144-SCO5145 intergenic		probable acyl CoA isomerase (SCO5144) conserved hypothetical protein (SCO5145)	B	B_r	B_r	N	B	N	N	B	dissociation	dissociation
74	SCO5331		possible DNA methylase	N	N	N	N	B	N	N	B		dissociation
75	SCO5639		hypothetical protein	B	B	B	N	B	N	N	B	dissociation	dissociation
76	SCO6499	*gvpO*	probable gas vesicle synthesis protein	N	N	N	N	B	N	N	B		dissociation
77	SCO6553-SCO6554 intergenic		probable integral membrane efflux protein probable transcriptional regulatory protein	N	N	N	N	B	N	N	B		dissociation
78	SCO7056		possible gntR-family transcriptional regulator	N	N	N	N	B	N	N	N		dissociation
79	SCO7073-SCO7074 intergenic		hypothetical protein possible membrane protein	B	N	N	N	B	N	N	B	dissociation	dissociation
80	SCO7763		possible membrane protein	N	N	N	N	B	N	N	B		dissociation

81	SCO1488-SCO1489 inte	*pyrR*	pyrimidine operon regulatory protein (SCO1488)	B	B	B	N	B	B_e	B_r	B_e		constant binding
	intergenic	*bldD*	putative DNA-binding protein (SCO1489)	B	B	B	N	B	B_e	B_r	B_e		constant binding
82	SCO1677		conserved hypothetical protein	N	N	N	N	B	B	B	B		constant binding
83	SCO3663-SCO3664		possible membrane protein (SCO3663)	N	N	N	N	B	B	B	B		constant binding
	intergenic		possible regulatory protein (SCO3664)										
84	SCO4010-SCO4011		possible secreted protein (SCO4010)	N	N	N	N	B	B	B	B		constant binding
	intergenic		possible integral membrane protein (SCO4011)										
85	SCO4075	*ragA*	ABC transport protein, ATP-binding subunit	N	N	N	N	B	B	B	B		constant binding
86	SCO4092	*hrpA*	ATP-dependent helicase<br>tRNA-Phe-Asp-Glu	N	N	N	N	B	B_e	B_e	B_r		constant binding
87	SCO4107		possible integral membrane protein<br>tRNA-Met	N	N	N	N	B	B	B	B		constant binding
88	SCO4635	*rmpG3*	50S ribosomal protein L33	B	B	B	B	B	B	B	B	constant binding	constant binding
89	SCO4646	*secE*	preprotein translocase SecE subunit	N	N	N	N	B	B	B	B		constant binding
90	SCO4987-SCO4988<br>intergenic		possible D-amino acid deaminase (SCO4987)<br>probable carbohydrate kinase (SCO4988)	N	N	N	N	B	B	B	B		constant binding
91	SCO5205		hypothetical protein	N	N	N	N	B	B	B	B		constant binding
92	SCO5549-SCO5550		tRNA-Gln-Glu	B	N	B	N	B	B	B	B	dissociation	constant binding
93	SCO1547		anthranilate synthase	N	N	N	N	N	B	B	N		induction of binding
94	SCO2461		possible secreted protein	N	N	N	N	B	B	B	B		constant binding
95	SCO3087		hypothetical	N	N	N	N	N	B	B	B		induction of binding
96	SCO3092		ncRNA	N	N	N	N	N	B	B	B		induction of binding
97	SCO3843		unknown	B	B	N	N	N	B	B	B	dissociation	induction of binding
98	SCO3945	*cydA*	cytochrome oxidase subunit I	N	N	N	N	N	B	B	N		induction of binding
99	SCO4067	*dnaZ*		N	N	N	N	N	B	B	B		induction of binding
100	SCO5579		possible transmembrane protein	B	B	B	B	N	N	N	N		
101	SCO5610-SCO5611<br>intergenic		unknown prophage gene (SCO5610)<br>possible prophage transcriptional regulator (SCO5611)	N	N	N	N	N	N	N	B		binding
102	SCO5705		hypothetical protein	N	N	N	N	N	B	B	N		binding
103	SCO6588-SCO6589<br>intergenic	 *fusB*	unknown (SCO6588)<br>possible alternative elongation fact or G	N	N	N	N	N	B	B	N		binding
104	SCO3138-SCO3139 inte	*galT*	galactose-1-phosphate uridylyltransferase<br>possible sodium:solute symporter (SCO3139)	N	N	N	N	B	N	N	B		dissociation
105	SCO3496		possible lyase precursor	N	N	N	N	N	N	N	B		binding
106	SCO5332		unknown	N	N	N	N	N	N	N	B		binding
107	SCO4123[d]		rrnA	B	N	B	N	N	B	B	B	dissociation	binding
108	SCO1792[d]		rrnB	B	N	B	N	N	B	B	B	dissociation	binding
109	SCO1390		rrnC	B	N	B	N	N	B	B	B	dissociation	binding
110	SCO5746		rrnE	B	N	B	N	N	B	B	B	dissociation	binding
111	SCO3334		rrnF	B	N	B	N	N	B	B	B	dissociation	binding

Dissociation refers to loss of binding after statistically relevant binding at an earlier time point. In some cases re-binding was observed but to avoid confusion this is also referreed to as "dissociation".
