# Supplementary material for: Genome-Wide Analysis of In Vivo Binding of the Master Regulator DasR in Streptomyces coelicolor Identifies Novel Non-Canonical Targets
Source: PLoS One. 2015 Apr 15;10(4):e0122479. doi: 10.1371/journal.pone.0122479 (PMC4398421; doi:10.1371/journal.pone.0122479)
Supplement: S3 Table — (PDF) [file pone.0122479.s008.pdf]

S3 Table. Genes whose expression was altered more than two-fold in the *dasR* null mutant as determined by microarray experiments.

Colour coding: blue, low expression; yellow, higher expression

	dasR/wt_24h	dasR/wt_30h	dasR/wt_36h	dasR/wt_42h	dasR/wt_54h	Function	Transcriptional regulators (nr)
SCO0019	-1,43	0,45	0,40	1,20	-1,10	hypothetical protein 19517:19771 reverse MW:9030	
SCO0022	0,07	0,21	0,17	1,27	0,95	putative IS element ATP-binding protein 22156:22299 reverse MW:5105	
SCO0098	-0,10	0,04	0,40	1,04	-0,13	putative transposase 81602:82441 reverse MW:31460	
SCO0107	1,00	0,85	-0,25	-0,40	-1,20	putative aminoglycoside nucleotidyltransferase 89486:90043 reverse MW:20294	
SCO0141	-0,24	-0,22	-0,38	-0,20	-1,25	putative calcium-binding protein 133570:134121 reverse MW:20507	
SCO0193	0,01	0,50	0,13	0,43	1,11	putative DNA-binding regulator 183793:184797 reverse MW:35255	1
SCO0194	-0,76	0,29	-0,21	0,48	1,24	putative sigma factor 184958:185566 forward MW:22300	2
SCO0195	-0,59	0,17	-0,26	0,27	1,21	putative lipoprotein 185675:186361 forward MW:22748	
SCO0196	-1,20	0,16	-0,11	0,44	0,61	hypothetical protein SCJ12.08 186483:186887 forward MW:14571	
SCO0381	0,02	1,06	0,73	0,92	0,28	putative glycosyl transferase 395939:397414 forward MW:53872	
SCO0383	-0,04	0,94	1,00	1,30	0,17	hypothetical protein SCF62.09 398881:400104 forward MW:45289	
SCO0396	-0,31	0,91	0,92	1,32	0,45	hypothetical protein SCF62.22 416973:418247 forward MW:46997	
SCO0399	0,08	0,86	0,74	1,23	0,48	putative membrane protein 420492:421715 forward MW:44113	
SCO0400	-0,18	0,88	0,67	1,09	0,36	putative epimerase 421712:422263 forward MW:19954	
SCO0494	0,94	0,26	0,19	1,03	-0,09	putative iron-siderophore binding lipoprotein 526786:527838 reverse MW:37375	
SCO0500	1,02	0,45	-0,54	0,50	-0,61	hypothetical protein 533534:533773 reverse MW:8377	
SCO0502	1,18	-1,47	0,02	-0,21	0,22	putative membrane protein 535974:536408 forward MW:15002	
SCO0527	1,43	-0,66	-0,01	-0,30	0,71	cold shock protein 558812:559015 reverse MW:7178	3
SCO0569	0,17	0,71	0,70	1,21	0,45	putative 50S ribosomal protein L36 (fragment) 611984:612106 reverse MW:4590	
SCO0591	-0,03	-0,40	-1,36	-0,64	0,98	putative lysozyme precursor 633119:633958 forward MW:30132	
SCO0644	0,92	0,09	0,97	1,37	1,18	putative membrane protein. 685288:687444 reverse MW:73843	
SCO0736	1,03	-0,13	1,30	0,57	0,89	putative secreted protein 779131:779826 reverse MW:25027	
SCO0850	-0,05	-0,33	-1,28	-0,28	0,52	putative membrane protein. 896543:896968 forward MW:14733	
SCO0923	0,10	-1,11	-0,05	0,00	-0,13	putative reductase flavoprotein subunit 968638:970587 reverse MW:72020	
SCO0930	1,01	0,34	1,44	1,78	1,41	putative lipoprotein 977491:978444 reverse MW:32028	
SCO0932	-1,42	-0,44	-0,35	0,19	-0,02	putative integral membrane protein 979340:980104 forward MW:27719	
SCO0933	0,13	-1,11	-0,28	-0,87	0,22	putative lipoprotein 980316:980834 forward MW:17455	
SCO0934	-0,15	-1,53	-2,11	-1,07	0,76	putative integral membrane protein 980917:981558 reverse MW:21435	
SCO0955	-0,19	-0,27	-0,63	0,10	1,06	hypothetical protein SCM11.10c 1004534:1004710 reverse MW:6386	
SCO0996	0,15	1,13	0,89	1,21	-0,42	putative lipoprotein 1051613:1052656 forward MW:37296	
SCO1050	0,13	-0,34	-1,03	-0,72	0,15	putative DNA protection protein 1106404:1107006 forward MW:22485	
SCO1121	1,15	0,19	1,33	1,79	1,23	putative secreted protein 1179094:1179834 forward MW:26309	
SCO1143	-0,19	-0,34	-1,40	-0,94	-0,65	conserved hypothetical protein 1201486:1202247 reverse MW:27112	
SCO1174	-1,99	0,14	0,06	-0,03	-0,12	aldehyde dehydrogenase 1237012:1238535 forward MW:55674	
SCO1189	0,00	0,42	0,71	1,09	0,83	hypothetical protein SCG11A.20 1262121:1262546 forward MW:15101	
SCO1192	-2,42	0,62	0,14	0,04	-0,41	hypothetical protein SCG11A.23 1264780:1265148 forward MW:13541	
SCO1195	-0,10	-0,37	-1,04	-0,51	-0,43	hypothetical protein SCG11A.26c 1267321:1267515 reverse MW:6553	
SCO1236	-0,01	-0,03	0,03	0,37	1,00	urease gamma subunit 1310293:1310595 reverse MW:11132	
SCO1240	-0,06	-0,07	-1,11	-0,34	0,73	putative NLP/P60 family protein 1312684:1313157 forward MW:16607	
SCO1255	0,61	-1,16	0,13	-0,47	-0,93	G/U mismatch-specific DNA glycosylase 1326802:1327284 forward MW:17531	

SCO1276	*sigJ*	-0,55	0,73	0,01	0,12	-1,63	RNA polymerase ECF sigma factor 1346693:1347289 forward MW:22530	4
SCO1288		-0,16	0,48	0,35	1,24	-0,66	putative integral membrane protein 1359227:1359733 reverse MW:18700	
SCO1321		1,21	0,48	0,26	0,17	-0,13	elongation factor TU-3 1396238:1397416 reverse MW:41676	
SCO1322		1,07	0,12	-0,05	0,06	-0,03	hypothetical protein 1397508:1398413 reverse MW:31669	
SCO1345		-1,09	-0,31	-0,33	-0,03	-0,19	putative short chain oxidoreductase 1421547:1422308 reverse MW:26271	
SCO1356		0,86	0,39	1,24	1,56	0,98	putative iron sulphur protein 1434218:1434715 forward MW:15473	
SCO1357		0,39	0,54	1,06	1,23	0,50	hypothetical protein 1434718:1435179 forward MW:17575	
SCO1415		0,25	-0,98	-1,36	-1,03	-0,15	putative secreted protein. 1510276:1510470 forward MW:6567	
SCO1426		-0,76	-0,40	-0,52	-0,55	-1,09	hypothetical protein SC6D7.13c. 1522328:1522765 forward MW:16128	
SCO1427		-1,83	-0,37	-0,59	-0,17	0,29	hypothetical protein SC6D7.12c 1522938:1523255 forward MW:11365	
SCO1474		-1,19	-0,44	-0,44	-0,34	-0,28	hypothetical protein SCL6.30 1574728:1575279 forward MW:19533	
SCO1480		0,93	-0,06	0,18	0,60	1,53	conserved hypothetical protein 1581512:1581835 reverse MW:11523	
SCO1505		0,95	-1,04	0,01	0,00	0,96	30S ribosomal protein S4 1608976:1609590 reverse MW:23579	
SCO1557		0,18	-0,08	1,19	0,60	0,28	putative lipoprotein 1669056:1669883 reverse MW:29013	
SCO1573		0,46	0,43	0,87	1,12	0,87	putative oxidoreductase 1683419:1684756 forward MW:49033	
SCO1575		0,22	0,28	0,89	1,21	0,65	thiamine biosynthesis lipoprotein precursor 1685643:1686383 MW:25098	
SCO1599		1,06	-1,09	-0,15	-0,17	1,18	50S ribosomal protein L35 1710812:1711006 reverse MW:7022	
SCO1600		1,42	-0,40	0,06	0,24	0,80	putative translation initiation factor IF-3 1711121:1711774 reverse MW:24149	
SCO1604		-0,74	1,21	0,46	1,16	-0,66	hypothetical protein SCI35.26c 1715151:1715678 reverse MW:19100	
SCO1659		-1,07	-0,51	-0,34	-0,30	0,02	putative glycerol uptake facilitator protein 1777840:1778634 forward MW:26820	
SCO1660		-1,53	-0,27	-0,16	-0,01	0,16	putative glycerol kinase 1778726:1780264 forward MW:55953	
SCO1674	*chpC*	-1,07	-1,09	-0,39	-0,27	0,44	putative secreted protein 1794330:1795109 reverse MW:24519	
SCO1675	*chpH*	-0,53	-2,23	-0,44	-0,39	1,39	putative small secreted protein 1795289:1795522 reverse MW:7344	
SCO1700		0,86	0,54	0,99	0,32	1,38	hypothetical protein 1820169:1820483 reverse MW:11289	
SCO1721		0,78	-0,85	-0,04	0,06	1,08	hypothetical protein 1841897:1842166 reverse MW:9831	
SCO1756		-1,03	-0,28	-0,54	-0,14	-0,31	hypothetical protein 2SCI34.09 1877577:1878374 forward MW:27638	
SCO1795		2,16	1,05	0,36	0,47	-0,35	hypothetical protein 1924446:1924952 forward MW:18850	
SCO1800	*chpE*	-1,73	-2,21	-1,25	-0,25	1,03	putative small secreted protein 1928593:1928841 reverse MW:7727	
SCO1828		-0,46	-1,27	-1,85	-0,88	0,18	hypothetical protein SCI8.13 1959189:1959422 forward MW:8351	
SCO1839		0,22	0,06	-0,70	0,17	1,43	putative transcriptional regulator 1967236:1967457 reverse MW:7637	
SCO1905		1,90	0,85	0,28	0,24	-0,18	hypothetical protein 2038879:2040480 reverse MW:58673	
SCO1914		0,77	-1,48	-0,21	-0,50	1,38	hypothetical protein 2049519:2049689 forward MW:6360	
SCO1978		0,52	-1,14	-0,07	-1,50	0,41	putative AbaA-like protein 2117630:2117905 reverse MW:9834	5
SCO1982		1,08	1,20	0,43	-0,22	-0,66	hypothetical protein 2120213:2120395 reverse MW:6459	
SCO2008		0,85	1,39	1,63	2,01	1,61	periplasmic branched chain amino acid binding protein 2149264:2150520 MW:43471	
SCO2012		0,44	0,90	1,28	1,64	1,04	branched chain amino acid transport ATP-binding protein 2154331:2155047 MW:25593	
SCO2113		-1,39	-1,51	-1,16	-0,84	0,20	bacterioferritin 2271144:2271647 forward MW:19219	
SCO2151		0,23	-1,02	-0,42	0,03	1,50	cytochrome c oxidase subunit III 2313623:2314243 reverse MW:22738	
SCO2155		-0,02	-0,57	-0,25	0,15	1,00	putative cytochrome c oxidase subunit I 2316758:2318494 reverse MW:64090	
SCO2156		0,90	-1,16	-0,21	-0,11	1,50	putative cytochrome c oxidase subunit II 2318491:2319450 reverse MW:35414	
SCO2195		-0,21	0,15	0,63	0,40	1,04	hypothetical protein 2363105:2363320 forward MW:7904	
SCO2205		0,08	1,26	1,67	1,65	0,24	hypothetical protein SC3H12.13c 2371009:2371323 reverse MW:11729	

SCO2207		1,45	1,03	1,57	1,80	0,93	hypothetical secreted protein 2371976:2372671 forward MW:23378	
SCO2210	*glnII*	0,98	0,49	0,72	0,91	1,47	glutamine synthetase 2374319:2375350 forward MW:37159	
SCO2217		1,15	0,15	1,62	2,48	2,45	putative secreted protein 2380490:2381995 forward MW:53672	
SCO2218		0,48	0,40	0,84	1,12	0,11	putative lipoprotein 2382072:2382518 reverse MW:14979	
SCO2236		1,20	0,47	-0,60	0,30	-0,21	conserved hypothetical protein 2406202:2406456 forward MW:9954	
SCO2261		-0,48	-0,70	-0,50	-1,71	-0,79	putative membrane protein. 2431192:2431449 reverse MW:8912	
SCO2288		-0,07	-0,08	-1,23	-0,48	0,20	hypothetical protein SCC75A.34c 2459004:2459330 reverse MW:12176	
SCO2379		1,44	0,90	0,27	0,49	-0,16	putative acetyltransferase 2549404:2549871 forward MW:16823	
SCO2389		0,07	-0,84	-0,68	-0,07	1,01	acyl carrier protein 2561443:2561691 forward MW:8917	
SCO2492		0,94	1,15	0,89	1,21	0,86	putative membrane protein 2682933:2683406 forward MW:15476	
SCO2496		0,73	0,77	0,90	1,28	0,29	putative secreted protein 2690016:2690456 forward MW:16154	
SCO2512		-0,32	0,12	-0,29	0,23	-1,22	hypothetical protein SCC121.15c 2709164:2709370 reverse MW:7138	
SCO2530		-1,06	-0,26	-0,31	-0,30	0,10	hypothetical protein SCC117.03. 2728656:2728922 forward MW:9588	
SCO2573		0,48	0,47	1,06	1,22	1,01	putative oxidoreductase. 2780292:2781866 forward MW:54599	
SCO2574		0,60	1,02	0,69	1,15	0,12	hypothetical protein SCC123.12c. 2782151:2782384 reverse MW:9015	
SCO2591		0,13	0,89	1,07	1,01	0,19	putative secreted protein 2803160:2804890 forward MW:61567	
SCO2597		0,74	-0,75	0,02	0,22	1,11	ribosomal protein L21 2812627:2812947 reverse MW:11632	
SCO2604		-0,43	1,13	0,45	0,99	-0,61	putative secreted protein 2825348:2826283 reverse MW:34315	
SCO2636		-0,43	0,01	-0,04	-0,74	-1,30	hypothetical protein SC8E4A.06 2864407:2865597 forward MW:42551	
SCO2658		-0,29	0,15	0,05	1,05	0,77	putative sugar-binding protein 2889601:2890938 forward MW:48299	
SCO2699	*chpG*	-0,83	-1,16	-1,57	-1,03	-0,30	putative small secreted protein 2943092:2943364 forward MW:8467	
SCO2705	*chpF*	-0,83	-2,30	-1,70	-0,93	0,59	putative secreted protein 2947104:2947370 reverse MW:8665	
SCO2716	*chpA*	-1,06	-1,48	-1,49	-0,87	-0,19	putative secreted protein 2960170:2960928 reverse MW:24640	
SCO2717	*chpD*	-1,30	-2,40	-0,86	-0,37	0,80	putative small secreted protein 2961104:2961331 reverse MW:7230	
SCO2718	*rdlA*	-1,57	-3,31	-1,30	-1,02	1,65	hypothetical protein SCC46.03c 2961619:2962014 reverse MW:13051	
SCO2719	*rdlB*	-1,79	-2,64	-1,17	-0,79	1,30	hypothetical protein SCC436.04 2962235:2962678 forward MW:14866	
SCO2739		-0,30	-0,02	-0,25	-0,04	-1,03	hypothetical protein SCC57A.10c. 2986899:2987096 reverse MW:7095	6
SCO2778		0,20	-1,16	-0,47	-0,46	-0,15	hydroxymethylglutaryl-CoA lyase 3031106:3032059 forward MW:33189	
SCO2789	*glmS2*	1,12	0,93	0,96	0,91	0,71	glucosamine-fructose-6-phosphate aminotransferase 3043786:3045603 MW:66117	
SCO2819		0,26	1,18	0,64	0,93	0,48	hypothetical protein 3079189:3079803 reverse MW:21680	
SCO2823		-0,07	-0,03	-0,13	0,48	1,31	putative decarboxylase 3083158:3084060 reverse MW:31221	
SCO2905	*nagF*	3,46	1,96	2,47	2,80	3,64	hypothetical protein 3157314:3157547 reverse MW:7904	
SCO2907	*nagE2*	1,62	1,06	1,71	2,11	1,65	putative PTS transmembrane component 3159228:3160478 forward MW:44168	
SCO2924	*ssgG*	-0,47	-1,10	-1,20	-1,93	-1,06	putative regulator 3175139:3175567 forward MW:15364	7
SCO2953		1,16	-0,03	1,00	2,00	1,21	putative membrane protein 3211305:3212093 reverse MW:27255	
SCO2954	*sigU*	0,77	0,53	1,11	1,71	1,44	putative RNA polymerase sigma factor 3212153:3212737 reverse MW:21718	8
SCO2976		0,26	0,91	0,70	1,21	1,35	hypothetical protein SCE50.04c 3240814:3241443 reverse MW:24276	
SCO3086		1,74	1,55	1,08	-0,93	0,45	putative lipoprotein 3379422:3380201 forward MW:26022	
SCO3092		0,55	-0,04	0,61	0,72	1,21	putative oxidoreductase (fragment) 3386512:3387852 reverse MW:48470	
SCO3105		1,36	1,16	0,65	0,17	-0,04	hypothetical protein SCE41.14 3401898:3402503 forward MW:20550	
SCO3106		1,28	0,92	0,57	0,10	-0,01	putative lipoprotein 3402553:3403452 forward MW:30430	
SCO3113		0,86	-0,43	-0,28	0,81	1,04	undefined product 3413129:3413194 forward MW:2092	

SCO3121		0,13	1,14	0,08	0,57	-1,00	hypothetical protein SCE41.30 3422070:3422198 forward MW:4498	
SCO3218		-1,56	0,11	-0,04	0,09	-0,54	putative small conserved hypothetical protein 3531249:3531464 reverse MW:7972	
SCO3220		-0,89	-0,37	-0,20	-0,54	-1,11	hypothetical protein 3532970:3533398 reverse MW:14729	
SCO3222		-2,17	-0,27	0,01	-0,06	-0,36	hypothetical protein 3534443:3534898 reverse MW:16320	
SCO3224		-1,11	0,17	-0,05	0,28	-1,20	putative ABC transporter ATP-binding protein 3535854:3536807 reverse MW:34090	
SCO3244		-1,15	-0,25	-0,26	-0,13	-0,76	hypothetical protein 3595842:3596639 reverse MW:29652	
SCO3262		-0,62	0,83	-0,27	0,30	-1,05	hypothetical protein 3612167:3612916 forward MW:27709	
SCO3263		2,96	2,59	4,76	5,53	3,99	hypothetical protein 3613331:3613630 reverse MW:10839	
SCO3265		2,12	2,17	3,25	3,45	2,81	hypothetical protein 3614367:3614591 reverse MW:8198	
SCO3266		0,51	0,93	1,33	1,77	0,61	hypothetical protein 3614588:3614791 reverse MW:7050	
SCO3268		2,31	1,83	3,45	4,08	4,16	hypothetical protein 3615121:3615399 reverse MW:9621	
SCO3269		-0,61	-0,03	-0,48	-0,21	-1,06	putative gntR-family regulator 3615479:3616234 forward MW:27363	9
SCO3271		-0,30	-0,37	-1,34	-0,34	-0,50	putative dehydrogenase 3617074:3617376 forward MW:11319	
SCO3278		0,00	-0,48	0,09	-1,12	-0,36	hypothetical protein 3622464:3622592 forward MW:4135	
SCO3299		2,48	0,42	0,31	0,17	-0,60	hypothetical protein 3647354:3648547 reverse MW:43187	
SCO3309		-0,55	-0,53	-0,14	-1,17	-0,52	hypothetical protein 3661177:3661554 reverse MW:13423	
SCO3323	*bldN*	0,29	0,01	0,01	0,73	1,35	putative RNA polymerase sigma factor 3675441:3675974 forward MW:20035	10
SCO3327		0,56	-0,91	-0,07	-0,06	1,56	hypothetical protein 3679847:3679945 reverse MW:4013	
SCO3343		0,60	0,31	1,02	0,76	-0,03	hypothetical protein 3700055:3700429 reverse MW:13021	
SCO3356	*sigE*	1,35	-0,12	0,83	0,06	1,00	ECF sigma factor 3715603:3716136 forward MW:20493	11
SCO3467		-0,41	0,61	0,48	1,11	-0,39	transposase 3826472:3827311 forward MW:31460	
SCO3475		-0,01	-0,65	-0,98	-1,26	-0,88	putative isomerase 3835869:3837002 reverse MW:40856	
SCO3476		-0,28	-0,29	-0,59	-0,93	-1,02	putative dehydrogenase 3837215:3837970 reverse MW:26202	
SCO3479		-0,28	-0,51	-0,66	-1,00	-0,77	putative beta-galatosidase 3840394:3843381 reverse MW:108825	
SCO3480		-0,15	-0,27	-0,64	-1,02	-0,84	putative racemase 3843522:3844607 reverse MW:39379	
SCO3481		0,11	-0,85	-0,98	-1,20	-0,90	hypothetical protein SCE65.17c 3844669:3845781 reverse MW:41112	
SCO3482		-0,40	-0,68	-0,94	-1,16	-0,53	putative sugar-permease 3845808:3846689 reverse MW:31611	
SCO3483		0,04	-1,12	-1,00	-1,30	-0,61	putative integral membrane transport protein 3846701:3847648 reverse MW:34520	
SCO3484		-0,28	-0,83	-1,18	-1,47	-1,24	putative secreted sugar-binding protein 3847684:3848994 reverse MW:46258	
SCO3487		-0,18	-1,57	-1,54	-1,76	-1,42	putative secreted hydrolase 3852248:3854644 forward MW:88595	
SCO3488		0,08	-0,81	-0,94	-1,42	-0,72	putative transposase remnant 3854813:3855157 forward MW:12854	
SCO3492		-0,12	-0,02	0,20	-0,70	-1,17	hypothetical protein SCE65.28c 3858302:3858430 reverse MW:4423	
SCO3549	*bldG*	0,54	-0,60	-0,04	0,39	1,01	putative anti-sigma factor antagonist 3924250:3924591 reverse MW:12289	12
SCO3561		-0,13	-1,07	-2,11	-1,48	-1,48	putative secreted protein 3936945:3938240 reverse MW:47117	
SCO3579	*wblA*	0,58	-0,16	0,01	0,08	1,13	putative regulatory protein 3957443:3957781 reverse MW:12595	13
SCO3607		-0,60	0,15	0,20	-0,01	-1,14	putative membrane protein 3983676:3985145 reverse MW:51612	
SCO3660		-1,01	0,08	-0,26	-0,26	-0,90	hypothetical protein 4038636:4040279 reverse MW:62365	
SCO3662		0,01	0,46	0,32	1,14	1,13	hypothetical protein SCH44.02c 4043329:4043664 reverse MW:12623	
SCO3663		-0,10	-1,15	-0,64	-0,73	0,20	putative secreted protein 4043666:4043857 reverse MW:6294	
SCO3671		0,78	-0,25	0,19	0,24	1,21	heat shock protein 70 (fragment) 4050836:4052692 reverse MW:66271	
SCO3720		-0,91	-0,24	1,08	0,48	-0,55	putative small membrane protein 4095827:4095910 reverse MW:2796	
SCO3731		1,28	-0,58	-0,26	-0,30	0,59	cold-shock protein 4105339:4105542 forward MW:7189	14

SCO3748		1,28	-0,44	-0,01	-0,24	1,04	cold shock protein 4118956:4119159 reverse MW:7189	15
SCO3762		-0,70	-0,72	-0,46	-0,45	-1,03	hypothetical protein SCH63.09c 4135075:4135272 reverse MW:6701	
SCO3793		0,82	1,14	1,20	0,12	0,18	conserved hypothetical protein 4171327:4171692 forward MW:13632	
SCO3863		0,37	-2,14	-1,65	-1,20	-0,50	hypothetical protein 4248266:4248655 forward MW:13116	
SCO3871		-0,32	-0,41	-1,41	-0,76	-0,63	putative decarboxylase 4258992:4259402 forward MW:15151	
SCO3899		0,08	-0,94	0,07	0,56	1,23	hypothetical protein 4293745:4294827 reverse MW:39229	
SCO3900		-0,03	-1,14	0,02	0,15	1,13	hypothetical protein 4294872:4295549 reverse MW:25185	
SCO3906		0,50	-1,07	0,08	-0,09	1,18	putative 30S ribosomal protein S6 4303241:4303531 forward MW:11177	
SCO3908		0,55	-0,94	0,31	0,09	1,06	putative 30S ribosomal protein S18 4304257:4304493 forward MW:8989	
SCO3909		0,42	-1,32	0,00	-0,01	1,06	putative 50S ribosomal protein L9 4304512:4304958 forward MW:15968	
SCO3918		-0,52	0,01	0,26	0,29	-1,40	hypothetical protein 4313835:4314317 reverse MW:17472	
SCO3982		4,08	3,87	5,16	6,00	5,55	conserved hypothetical protein 4384450:4384728 forward MW:9848	
SCO3983		3,46	2,79	4,08	4,53	4,60	hypothetical protein 4384741:4385052 forward MW:11416	
SCO3984		1,79	2,02	2,68	2,92	2,44	conserved hypothetical protein 4385052:4385255 forward MW:7053	
SCO3985		3,05	3,09	4,26	4,84	4,19	conserved hypothetical protein 4385252:4385476 forward MW:7934	
SCO3986		2,80	1,95	3,61	3,94	3,67	putative GntR-family transcriptional regulator 4385485:4386216 forward MW:26537	16
SCO3987		3,28	2,99	3,93	4,51	4,16	conserved hypothetical protein 4386216:4386521 forward MW:10828	
SCO3988		2,06	1,61	2,25	2,67	2,49	hypothetical protein 4386551:4386898 forward MW:12451	
SCO3989		-0,52	0,65	0,55	1,10	-0,32	putative ribonuclease inhibitor 4387239:4387508 forward MW:10195	
SCO3990		-0,47	0,69	0,45	1,00	-1,22	hypothetical protein 4387790:4388059 reverse MW:9816	
SCO4002		-0,93	-0,74	-1,03	-0,80	-1,44	putative secreted protein 4396911:4397237 forward MW:10413	
SCO4032		-0,17	-1,18	-0,55	-0,60	-0,35	putative marR regulatory protein 4429661:4430161 reverse MW:18359	17
SCO4057		-0,12	-1,06	-1,52	-1,30	-0,63	putative transport integral membrane protein 4449454:4450344 reverse MW:31873	
SCO4063		-0,23	0,39	0,66	1,29	-0,59	putative secreted protein 4453498:4453854 reverse MW:13015	
SCO4091		0,37	0,22	0,19	0,47	1,27	putative DNA-binding protein 4485362:4485568 forward MW:7545	18
SCO4177		1,47	0,15	0,08	0,47	0,68	conserved hypothetical protein 4589531:4589800 forward MW:9451	19
SCO4178		1,81	0,36	0,25	0,58	0,95	hypothetical protein SCD66.15 4589845:4589943 forward MW:3525	
SCO4187		-0,57	0,98	0,50	0,16	1,54	hypothetical protein 4597039:4597224 reverse MW:6268	
SCO4198		-0,39	-1,24	-0,75	-0,43	0,75	putative DNA-binding protein 4607361:4607786 forward MW:15476	20
SCO4212		0,25	-1,86	-0,08	-1,20	0,73	hypothetical protein 4620304:4620666 forward MW:12897	
SCO4213		-0,58	0,11	0,08	0,22	-1,14	hypothetical protein 4621090:4622724 forward MW:61188	
SCO4216		0,30	0,81	0,33	0,74	-1,14	hypothetical protein 4624586:4624795 reverse MW:7745	
SCO4239		-0,98	-1,07	-0,78	-0,07	-0,18	putative secreted protein 4644505:4644951 reverse MW:15668	
SCO4251		-1,34	-0,29	-0,28	-0,01	-0,11	putative secreted protein 4660279:4660812 reverse MW:18848	
SCO4252		-1,79	0,11	0,18	0,72	-0,13	hypothetical protein SCD8A.25c 4660812:4661261 reverse MW:16480	
SCO4266		-0,26	-0,55	-1,28	0,26	0,19	putative oxidoreductase 4681885:4682865 reverse MW:33006	
SCO4285	*nagK*	1,24	0,79	1,46	1,59	1,50	putative sugar kinase 4700542:4701522 reverse MW:32584	
SCO4289		1,11	0,72	0,92	-1,04	-0,16	putative secreted protein 4704589:4705071 forward MW:15547	
SCO4296		0,27	-0,41	0,02	1,04	1,38	chaperonin 2 4711114:4712739 forward MW:56830	
SCO4442		0,32	-0,49	0,12	0,19	1,91	hypothetical protein SCD6.20 4864680:4864871 forward MW:6484	21
SCO4516		-0,30	1,12	0,26	0,61	-0,87	hypothetical protein SCD35.23c 4938160:4938519 reverse MW:13763	
SCO4540		-0,50	1,20	-0,45	0,15	-1,33	hypothetical protein 2SCD4.11c 4957301:4957543 reverse MW:8865	

SCO4543	-1,01	-0,13	-0,21	-0,05	-0,35	hypothetical protein 4959583:4960434 reverse MW:31767
SCO4562	-0,73	-0,39	-1,08	-0,40	0,00	nuoA NADH dehydrogenase subunit 4980273:4980632 forward MW:13223
SCO4611	-0,04	0,38	0,65	0,72	1,06	hypothetical protein SCD39.11 5035357:5035950 forward MW:21390
SCO4630	1,63	1,59	1,11	1,44	0,39	hypothetical protein SCD82.01c 5051464:5052723 reverse MW:46689
SCO4635	2,37	-0,03	0,48	0,42	2,03	50S ribosomal protein L33 5061598:5061762 forward MW:6374
SCO4636	0,73	0,87	0,61	0,70	1,38	hypothetical protein SCD82.07 5061858:5062310 forward MW:16152
SCO4642	0,62	-0,23	0,25	0,30	1,19	hypothetical protein SCD82.13 5066623:5067003 forward MW:14127
SCO4648	1,49	-0,48	0,53	0,64	2,09	50S ribosomal protein L11 5072850:5073284 forward MW:15394
SCO4649	1,33	-0,88	0,17	0,38	1,96	50S ribosomal protein L1 5073368:5074093 forward MW:25731
SCO4651	1,21	1,17	0,77	0,44	0,30	putative lipoprotein 5075272:5076120 forward MW:30048
SCO4652	1,00	-1,84	-0,02	-0,09	1,89	50S ribosomal protein L10 5076439:5076969 forward MW:18646
SCO4653	0,51	-1,05	0,00	0,20	1,06	50S ribosomal protein L7/L12 5077087:5077470 forward MW:13209
SCO4659	1,30	-1,08	-0,05	-0,09	1,40	30S ribosomal protein S12 5088945:5089316 forward MW:13739
SCO4660	1,27	-0,83	-0,07	0,02	1,27	30S ribosomal protein S7 5089319:5089789 forward MW:17431
SCO4662	1,08	-0,79	0,14	0,42	1,99	elongation factor TU-1 5092129:5093322 forward MW:43749
SCO4663	0,94	0,13	0,54	0,84	1,08	hypothetical protein SCD40A.09c 5093312:5093665 reverse MW:12538
SCO4677	0,68	1,18	0,78	0,99	1,54	putative regulatory protein 5108376:5108810 reverse MW:15858
SCO4687	0,72	0,53	0,39	1,06	1,13	hypothetical protein SCD31.12c 5115960:5116319 reverse MW:12901
SCO4701	1,65	-0,79	0,13	0,18	1,75	30S ribosomal protein S10 5127608:5127916 forward MW:11521
SCO4702	1,41	-0,98	0,18	0,02	1,82	50S ribosomal protein L3 5127933:5128577 forward MW:22769
SCO4703	0,81	-0,76	0,02	0,02	1,29	50S ribosomal protein L4 5128586:5129245 forward MW:23643
SCO4704	1,39	-1,04	-0,03	0,20	1,84	50S ribosomal protein L23 5129245:5129664 forward MW:14978
SCO4705	1,23	-0,96	-0,09	0,05	1,54	50S ribosomal protein L2 5129705:5130541 forward MW:30551
SCO4706	1,53	-1,11	0,07	0,03	1,87	30S ribosomal protein S19 5130554:5130835 forward MW:10573
SCO4707	1,79	-0,89	-0,11	0,15	1,66	50S ribosomal protein L22 5130848:5131225 forward MW:13863
SCO4708	0,88	-0,78	0,21	0,15	1,35	30S ribosomal protein S3 5131225:5132058 forward MW:30273
SCO4709	1,15	-0,83	0,33	0,30	1,58	50S ribosomal protein L16 5132064:5132483 forward MW:15843
SCO4711	1,53	-1,49	0,01	0,15	2,08	30S ribosomal protein S17 5132707:5132994 forward MW:10720
SCO4712	1,00	-1,30	0,10	0,06	1,71	50S ribosomal protein L14 5133102:5133470 forward MW:13337
SCO4713	1,15	-1,15	-0,05	0,12	1,17	50S ribosomal protein L24 5133473:5133796 forward MW:11590
SCO4714	0,98	-1,48	-0,03	-0,08	1,67	50S ribosomal protein L5 5133796:5134353 forward MW:20853
SCO4715	0,81	-1,37	-0,14	-0,10	1,57	30S ribosomal protein S14 5134359:5134544 forward MW:6949
SCO4716	0,88	-0,97	0,05	0,13	1,60	30S ribosomal protein S8 5134756:5135154 forward MW:14276
SCO4717	1,09	-1,15	0,07	0,04	1,77	50S ribosomal protein L6 5135179:5135718 forward MW:19179
SCO4718	1,03	-1,03	0,18	0,16	1,52	50S ribosomal protein L18 5135722:5136105 forward MW:13578
SCO4719	0,47	-0,75	0,04	0,17	1,03	30S ribosomal protein S5 5136149:5136754 forward MW:20499
SCO4720	0,83	-1,68	-0,09	-0,01	2,21	50S ribosomal protein L30 5136757:5136939 forward MW:6888
SCO4721	0,81	-1,66	0,00	-0,11	1,92	50S ribosomal protein L15 5136942:5137397 forward MW:15947
SCO4723	0,73	0,53	0,77	0,78	1,07	adenylate kinase (fragment) 5138967:5139620 forward MW:23985
SCO4725	1,63	-0,88	0,44	0,19	2,12	translational initiation factor IF1 5140750:5140971 forward MW:8378
SCO4727	1,19	-0,91	0,00	0,02	1,37	30S ribosomal protein S13 5141342:5141722 forward MW:14219
SCO4728	1,09	-1,01	0,20	0,15	1,44	30S ribosomal protein S11 5141790:5142194 forward MW:14398

SCO4729		0,95	-0,86	0,38	0,02	1,14	DNA-directed RNA polymerase alpha chain 5142326:5143348 forward MW:36696	
SCO4730		1,19	-0,86	0,27	0,27	1,26	50S ribosomal protein L17 5143543:5144049 forward MW:18130	
SCO4734		1,59	0,13	0,28	0,45	1,18	50S ribosomal protein L13 5147450:5147893 forward MW:16416	
SCO4735		1,09	0,15	0,34	0,26	0,91	30S ribosomal protein S9 5147936:5148448 forward MW:18667	
SCO4740	*glmS1*	0,42	-0,02	0,04	0,37	1,36	glucosamine--fructose-6-phosphate aminotransfera se 5153333:5155180 MW:65561	
SCO4756		0,18	0,50	0,51	1,07	0,57	undefined product 5167796:5168152 forward MW:13246	
SCO4759		0,00	-0,06	-1,02	-0,28	-0,28	putative secreted protein 5170779:5171654 forward MW:32608	
SCO4761		0,99	-0,91	0,04	1,32	2,32	10 kD chaperonin cpn10 5172736:5173044 forward MW:10914	
SCO4762		0,22	-0,25	0,41	1,36	1,37	60 kD chaperonin cpn60 5173178:5174803 forward MW:57119	
SCO4770		0,79	0,50	0,80	1,01	0,97	inosine 5' monophosphate dehydrogenase 5181346:5182851 forward MW:52476	
SCO4779		1,33	0,85	1,04	-0,17	0,44	serine/threonine protein kinase 5197709:5199355 forward MW:58091	
SCO4789		0,93	0,70	1,15	1,49	1,26	putative integral membrane protein 5209774:5210289 forward MW:18294	
SCO4794		0,61	0,42	0,64	1,02	0,55	putative integral membrane protein 5215427:5215963 forward MW:18436	
SCO4800		0,36	0,67	1,00	0,64	1,15	isobutiryl CoA mutase small subunit 5224491:5224907 forward MW:14314	
SCO4808		0,71	-0,45	0,67	0,76	2,00	succinyl-CoA synthetase beta chain 5234992:5236176 forward MW:41570	
SCO4809		0,39	-0,39	0,40	0,58	1,54	succinyl CoA synthetase alpha chain 5236205:5237089 forward MW:30239	
SCO4823		0,30	1,05	-0,22	0,90	0,19	hypothetical protein SC2A6.08 5253807:5253983 forward MW:6376	
SCO4838		0,52	-1,09	-0,20	-0,56	0,58	hypothetical protein 5270745:5271401 reverse MW:23490	
SCO4839		0,74	0,29	0,65	0,58	1,13	tryptophanyl-tRNA synthetase 5271518:5272537 forward MW:37593	
SCO4840		1,05	0,56	0,37	0,66	0,95	putative secreted protein 5272714:5273292 forward MW:20500	
SCO4854		0,68	0,30	0,85	1,12	1,70	putative integral membrane protein 5283652:5284272 reverse MW:22056	
SCO4855		1,05	0,52	0,60	0,57	0,98	succinate dehydrogenase iron-sulfur subunit 5284438:5285211 reverse MW:29301	
SCO4856		1,25	0,45	0,55	0,78	1,04	succinate dehydrogenase flavoprotein subunit 5285211:5286965 reverse MW:64788	
SCO4858		0,99	0,55	0,49	0,77	1,36	succinate dehydrogenase membrane subunit 5287473:5287853 reverse MW:14076	
SCO4861		0,32	1,31	1,21	1,70	0,68	hypothetical protein SCK20.02 5290561:5291106 forward MW:18889	
SCO4883		1,15	0,79	0,80	0,95	1,61	putative peptidase 5315651:5316883 forward MW:43698	
SCO4884		0,30	0,98	1,15	0,62	0,46	putative lipoprotein 5317103:5318146 forward MW:35708	
SCO4894		0,81	1,05	1,24	1,98	0,89	hypothetical protein 2SCK8.20c 5328162:5328413 reverse MW:8617	
SCO4895		0,53	0,85	0,92	1,04	0,73	putative ECF sigma factor 5328417:5329421 reverse MW:36822	22
SCO4902		-1,48	-1,15	-0,50	-0,12	0,84	putative secreted protein 5335876:5336244 forward MW:11834	
SCO4903		0,70	0,55	0,61	1,39	0,74	putative membrane protein 5336332:5336535 reverse MW:7275	
SCO4904		1,32	1,13	0,97	-0,13	0,43	putative integral membrane protein 5336631:5337182 reverse MW:19055	
SCO4908	*sigQ*	1,16	1,07	0,73	-0,42	1,35	putative RNA polymerase sigma factor (fragment) 5340551:5341141 forward MW:224	23
SCO4920		0,31	0,71	0,64	0,74	1,38	putative deoR-family transcriptional regulator 5354228:5355181 forward MW:32330	24
SCO4968		0,59	-0,45	0,10	0,35	1,41	putative membrane protein 5402116:5402343 forward MW:7987	
SCO5029		0,75	-0,69	0,36	0,42	1,86	putative secreted protein 5464766:5465482 forward MW:25183	
SCO5071		-0,45	-0,27	-0,47	1,23	0,30	hydroxylacyl-CoA dehydrogenase 5513809:5514249 reverse MW:16173	
SCO5074		-1,04	-0,57	-0,36	1,52	0,84	hypothetical protein 5516299:5516943 forward MW:23134	
SCO5075		-0,26	-0,26	-0,24	1,07	0,45	putative oxidoreductase (fragment) 5516977:5517897 forward MW:31486	
SCO5078		-0,37	-0,34	-0,09	1,18	0,35	hypothetical protein 5519987:5520832 forward MW:30029	
SCO5082		-0,24	-0,58	-0,18	1,14	1,34	putative transcriptional regulatory protein 5523183:5523962 reverse MW:28236	25
SCO5083		-0,07	-0,86	-0,49	1,14	0,89	putative actinorhodin transporter 5524073:5525809 forward MW:59772	

SCO5084		-0,02	-1,23	-0,49	1,48	1,22	putative membrane protein 5525809:5527944 forward MW:74863	
SCO5085	*actII-4*	-0,89	-0,48	-0,06	1,31	0,77	actinorhodin cluster activator protein 5528094:5528861 forward MW:28763	26
SCO5089		-0,40	-0,33	-0,45	1,04	0,36	actinorhodin polyketide synthase acyl carrier protein 5532449:5532709 forward MW:9248	
SCO5112	*bldKa*	0,23	0,64	0,46	0,85	1,06	BldKA ABC transport system integral membrane protein 5557355:5558386 MW:37471	
SCO5113	*bldKb*	0,04	0,61	0,66	1,00	1,16	BldKB ABC transport system lipoprotein 5558498:5560300 forward MW:65533	
SCO5137		-1,06	-0,49	-0,39	-0,44	-0,23	putative ATP-binding protein 5584655:5585086 reverse MW:12978	
SCO5163		0,25	0,08	0,10	0,12	1,11	hypothetical protein SCP8.26c 5609892:5610044 reverse MW:5470	
SCO5189		2,22	0,75	0,13	0,10	0,12	hypothetical protein 2SC3B6.13 5647169:5647570 forward MW:14131	
SCO5190	*wblC*	3,31	0,82	-0,10	0,35	-0,39	putative DNA-binding protein 5647747:5648115 forward MW:13169	27
SCO5191		3,15	0,22	0,31	0,35	-0,56	hypothetical protein 2SC3B6.15 5648112:5648435 forward MW:12266	
SCO5232	*dasA*	1,38	1,43	1,47	1,58	0,64	putative sugar transporter sugar binding protein 5692292:5693569 forward MW:45260	
SCO5233	*dasB*	0,49	0,39	0,78	1,11	0,29	putative sugar transporter integral membrane protein 5693658:5694644 forward MW:36132	
SCO5234	*dasC*	0,44	0,41	1,10	1,11	0,29	putative sugar transporter integral membrane protein 5694641:5695471 forward MW:30648	
SCO5236	*nagB*	1,89	1,48	2,04	2,06	2,01	putative glucosamine phosphate isomerase 5697291:5698076 reverse MW:27414	
SCO5249		-1,46	-0,77	-0,91	-0,68	-0,25	putative nucleotide-binding protein 5709624:5711030 forward MW:52193	28
SCO5272		-0,55	-0,79	-0,53	-1,55	-0,60	hypothetical protein 2SC7G11.34 5734620:5734814 forward MW:6919	
SCO5276		-0,52	-0,06	-0,53	-0,36	-1,14	conserved hypothetical protein 5741653:5742078 forward MW:14540	
SCO5314	*whiE* ORF7	0,36	-0,15	-0,93	-0,53	1,04	whiE protein VII 5785753:5786088 reverse MW:12407	
SCO5316	*whiE* ORF5	0,01	-0,18	-0,99	-0,66	1,22	acyl carrier protein 5786603:5786875 reverse MW:9750	
SCO5319	*whiE* ORFII	0,00	-1,27	-1,18	-0,46	0,69	whiE protein II 5789484:5789957 reverse MW:16669	
SCO5326		-0,13	0,02	0,48	1,46	-0,97	hypothetical protein 5796324:5796578 reverse MW:8786	
SCO5329		-0,61	1,44	0,51	1,05	-0,66	hypothetical protein 5798773:5800470 forward MW:64473	
SCO5332		-0,47	1,24	0,41	0,38	-1,64	hypothetical protein SCBAC5H2.01 (fragment) 5805823:5807334 forward MW:56610	
SCO5389		-2,36	-0,27	-0,54	0,02	-0,37	hypothetical protein 5858397:5858789 forward MW:14303	
SCO5476		0,50	0,94	1,13	1,15	0,65	oligopeptide transport integral membrane protein 5962095:5963102 forward MW:35744	
SCO5477		0,91	1,01	0,85	1,40	1,10	putative oligopeptide-binding lipoprotein 5963174:5964976 forward MW:65282	
SCO5478		0,69	0,80	0,99	1,12	0,68	oligopeptide transport system integral membrane protein 5965085:5966083 MW:35796	
SCO5498	*gatC*	0,57	0,10	0,21	0,46	1,06	probable Glu-tRNAGln amidotransferase subunit C 5984816:5985112 MW:10779	
SCO5521		1,04	1,01	0,70	1,05	0,86	hypothetical protein SC1C2.02 6015052:6015183 forward MW:4791	
SCO5542		0,18	0,48	0,84	1,35	0,66	hypothetical protein SC1C2.23c 6039645:6040043 reverse MW:14089	
SCO5556		-0,13	-1,60	-1,66	-0,77	0,30	histone-like DNA binding protein 6054696:6055352 forward MW:22316	
SCO5571		1,07	-1,14	-0,07	-0,07	1,29	50S ribosomal protein L32 6069794:6069967 forward MW:6540	
SCO5576		-0,11	-0,03	-0,09	-0,15	-1,03	putative acylphosphatase 6073650:6073931 forward MW:10308	
SCO5582	*nsdA*	0,04	0,07	-0,01	0,19	1,13	putative transcriptional regulator 6084219:6085721 forward MW:55068	29
SCO5583	*amtB*	0,31	0,13	0,43	0,68	1,23	ammonium transporter 6086031:6087377 forward MW:46323	
SCO5584	*glnK*	0,55	0,05	0,21	0,50	1,16	nitrogen regulatory protein pII 6087374:6087712 forward MW:12232	30
SCO5590		-0,42	-0,16	0,03	0,16	1,01	hypothetical protein SC2E1.07 6096339:6096935 forward MW:21105	
SCO5591		0,93	-0,74	0,34	0,03	1,44	30S ribosomal protein S16 6097191:6097610 forward MW:15164	
SCO5592		1,30	-0,40	-0,09	0,23	1,33	hypothetical protein SC2E1.09 6097613:6097852 forward MW:8683	
SCO5609		-1,34	-0,61	-0,82	-0,98	-1,21	hypothetical protein SC2E1.26c 6109515:6109712 reverse MW:6351	
SCO5610		1,17	1,74	0,65	0,58	-0,38	hypothetical protein SC2E1.27c 6110051:6110413 reverse MW:13093	
SCO5611		0,10	0,72	0,42	1,48	-0,77	putative transcriptional regulator 6111177:6111392 forward MW:7863	31

SCO5614		-0,86	0,55	0,10	1,20	-0,98	putative transcriptional regulator 6115124:6115315 forward MW:6962	32
SCO5632		0,16	1,39	1,26	1,59	2,61	hypothetical protein 6132437:6132679 reverse MW:8201	
SCO5638		-0,91	0,82	-0,37	0,18	-1,45	integral membrane protein 6137881:6138768 reverse MW:32780	
SCO5639		-1,29	0,82	0,10	0,94	-1,34	hypothetical protein SC6A9.28 6138765:6139295 reverse MW:20433	
SCO5640		-0,87	0,66	-0,49	0,11	-1,25	hypothetical protein SC6A9.27 6139402:6139869 reverse MW:17110	
SCO5650		0,54	0,23	0,41	0,57	1,03	hypothetical protein SC6A9.17 6149064:6149768 reverse MW:23571	
SCO5672		-0,20	-1,00	-0,69	-1,35	-0,80	undefined product 6172204:6172662 forward MW:15731	
SCO5705		1,08	-0,24	-0,08	0,03	0,83	hypothetical protein 6215999:6216304 forward MW:10995	
SCO5736		0,85	-0,83	0,00	0,05	1,44	30S ribosomal protein S15 6259203:6259490 forward MW:10758	
SCO5789		-1,80	-0,49	-0,59	0,15	0,04	hypothetical protein SC4H2.10c 6326868:6327161 reverse MW:9900	
SCO5796		1,05	0,63	0,28	-0,01	-0,05	hypothetical protein SC4H2.17 6334502:6335995 forward MW:54507	
SCO5826		-1,17	0,05	-0,11	0,70	0,92	undefined product 6375779:6376357 forward MW:20623	
SCO5841	*ptsH*	1,51	1,30	1,18	1,23	1,59	phosphocarrier protein hpr 6394045:6394326 reverse MW:9390	
SCO5885		-0,80	0,18	0,91	1,02	0,04	hypothetical protein SC3F7.05c 6442174:6442614 reverse MW:15637	
SCO5920		0,98	1,29	0,16	-1,14	-0,48	undefined product 6487772:6489268 reverse MW:52678	
SCO5990		-0,18	-0,55	-1,13	-0,82	-0,58	hypothetical protein 6564792:6565028 reverse MW:8721	
SCO6005	*ngcE*	1,07	1,23	1,45	2,10	1,98	hypothetical protein SC7B7.02 6584624:6586033 forward MW:50611	
SCO6006	*ngcF*	0,60	0,94	1,11	1,39	1,47	probable transmembrane transport protein 6586041:6587102 forward MW:38014	
SCO6007	*ngcG*	0,78	0,85	1,35	1,84	1,27	probable transmembrane transport protein 6587099:6588007 forward MW:33732	
SCO6027		-1,24	-0,51	-0,23	-0,72	-0,29	acetyl-coa acetyltransferase (thiolase) 6615297:6616514 reverse MW:43132	
SCO6029	*whiI*	0,08	-1,00	-0,83	-0,50	0,26	two-component regulator 6618177:6618839 reverse MW:22747	33
SCO6042		0,09	-0,24	0,14	0,44	1,07	hypothetical protein SC1B5.02 6633983:6634714 forward MW:28132	
SCO6149		1,86	1,15	0,43	0,43	-0,21	putative integral membrane nucleotide binding protein 6750131:6751231 MW:38706	
SCO6197		0,35	1,56	1,57	2,41	0,67	putative secreted protein 6807680:6808222 reverse MW:19395	
SCO6240		0,90	1,35	0,08	0,79	-0,61	hypothetical protein SCAH10.05c 6863358:6864092 reverse MW:25783	
SCO6266	*scbA*	1,10	0,28	0,32	0,54	0,00	ScbA protein 6891293:6892237 forward MW:33747	
SCO6276		-1,07	0,91	0,10	0,25	0,05	putative secreted protein 6932285:6933607 forward MW:48024	
SCO6279		-1,02	1,03	0,13	0,27	0,10	putative diaminobutyrate-pyruvate aminotransferase 6936149:6937705 MW:56863	
SCO6280	*cpkO*	0,99	0,08	0,28	-0,07	0,38		
SCO6282		-1,59	0,53	0,29	0,25	0,86	putative oxidoreductase 6941746:6942543 reverse MW:27431	
SCO6283		-1,05	0,10	0,05	0,18	0,07	conserved hypothetical protein 6942661:6943506 forward MW:29783	
SCO6338		-0,19	0,11	0,39	1,35	-0,89	putative transposase 6997411:6997698 reverse MW:10607	
SCO6373		-0,29	-0,09	-1,10	-0,39	-0,35	putative integral membrane protein 7034266:7035663 reverse MW:50082	
SCO6374		-0,03	-0,16	-1,07	-0,45	-0,60	putative sugar transferase 7035788:7037224 reverse MW:52310	
SCO6401		-0,31	1,09	0,33	1,00	-0,66	hypothetical protein SC3C8.21c 7067605:7068132 reverse MW:19100	
SCO6433		-0,45	0,62	0,62	1,50	0,10	hypothetical protein 7116086:7116493 forward MW:15025	
SCO6440		-0,04	-0,02	-0,29	-0,33	-1,04	conserved hypothetical protein 7123131:7123883 forward MW:26158	
SCO6441		-0,10	0,00	-0,50	-0,35	-1,12	putative aldehyde dehydrogenase 7123886:7125292 forward MW:48948	
SCO6543		-0,25	-0,02	-0,28	0,16	-1,02	hypothetical protein SC5C7.28c 7236695:7236940 reverse MW:9004	
SCO6592		0,46	0,98	1,04	0,86	-0,62	putative secreted protein 7306230:7306619 reverse MW:13707	
SCO6624		-0,68	-1,64	-0,58	0,41	1,53	hypothetical protein SC1F2.21c 7347312:7347440 reverse MW:4641	
SCO6631		0,15	1,02	0,69	0,97	0,64	hypothetical protein SC4G2.05 7358814:7359689 forward MW:30980	

SCO6650		1,41	0,13	1,79	2,46	2,75	hypothetical protein SC4G2.24 7385660:7386058 forward MW:14383	
SCO6652		0,56	0,44	1,02	1,08	0,85	hypothetical protein SC5A7.02 7387290:7388675 forward MW:47141	
SCO6655		0,66	0,46	1,34	2,02	1,38	hypothetical protein SC5A7.05 7390536:7391201 forward MW:24086	
SCO6682	*ramS*	-4,44	-3,13	-2,72	-1,73	-0,77	hypothetical protein SC5A7.32 7422494:7422622 forward MW:4496	
SCO6715		0,40	1,42	-0,38	-1,92	-0,80	putative transcriptional regulator 7469875:7470120 forward MW:8989	34
SCO6736		0,09	-0,02	1,04	0,09	-0,45	putative metallopeptidase 7490559:7492079 reverse MW:54883	
SCO6810		-0,13	-1,14	-0,38	-0,76	0,29	hypothetical protein SC1A2.19c. 7572343:7572801 reverse MW:16226	
SCO6812		0,37	1,42	0,20	0,10	-0,43	putative ArsR-family transcriptional regulator. 7574420:7574779 forward MW:12805	35
SCO6836		-1,27	0,01	0,40	-0,17	0,29	putative ArsR-family transcriptional regulator. 7606744:7607052 forward MW:11257	36
SCO6906		-0,14	1,21	0,37	1,05	-0,14	hypothetical protein SC1B2.12c. 7669618:7671207 reverse MW:57434	
SCO6909		-0,46	0,56	-0,06	-0,24	-1,29	hypothetical protein SC1B2.15. 7674795:7675178 forward MW:14695	
SCO6910		-0,52	0,49	0,24	1,29	-0,60	insertion element transposase. 7675634:7676473 reverse MW:31460	
SCO6952		1,05	0,62	0,05	0,06	-0,52	hypothetical protein SC6F7.05c. 7715801:7716844 reverse MW:38991	
SCO7106	*wblJ*	0,02	-1,05	-0,72	-0,89	-0,52	conserved hypothetical protein 7896424:7896690 forward MW:9550	37
SCO7190		-0,23	0,85	0,36	1,02	-0,21	hypothetical protein 7993470:7994357 forward MW:33452	
SCO7195		-0,13	0,73	0,11	0,56	-1,05	hypothetical protein SC8A11.23c 7999105:7999371 reverse MW:9851	
SCO7252	*nsdB*	-0,44	0,24	0,28	0,65	1,31	putative regulatory protein 8062135:8063643 forward MW:54823	38
SCO7257	*chpB*	-1,18	-0,48	-0,45	0,22	0,20	hypothetical protein 8069445:8070158 forward MW:22741	
SCO7336		-0,19	-0,21	-2,00	-0,99	0,23	hypothetical protein. 8150499:8150771 reverse MW:10000	
SCO7387		0,24	-1,63	-0,16	-0,30	0,14	hypothetical protein SC10G8.14. 8199503:8200009 forward MW:18283	
SCO7434		0,11	-0,91	-1,92	-1,48	-0,65	putative lipoprotein. 8247219:8247980 forward MW:26600	
SCO7449		0,21	-1,12	-1,91	-1,28	0,83	putative membrane protein. 8264413:8265090 forward MW:22745	
SCO7452		-0,12	-1,20	-1,40	-1,17	-0,03	putative O-methyltransferase. 8267283:8268311 reverse MW:37035	
SCO7453		-0,10	-2,05	-1,91	-1,33	0,07	hypothetical protein SC5C11.10c. 8268376:8269446 reverse MW:36624	
SCO7536		0,04	0,51	1,11	1,15	0,19	putative integral membrane protein. 8355539:8357758 forward MW:76089	
SCO7643		0,21	-0,04	0,12	1,09	-0,43	hypothetical protein SC10F4.16 8471548:8471823 forward MW:10066	
SCO7657		-0,25	-0,38	0,38	1,26	0,96	putative secreted protein 8483781:8485538 forward MW:61726	
SCO7658		-0,01	-0,05	0,92	1,55	0,96	hypothetical protein SC10F4.31 8485558:8485710 forward MW:5511	
SCO7676		-0,46	-1,05	-0,58	-0,70	-0,48	putative ferredoxin 8499061:8499282 forward MW:7430	
SCO7699		-1,09	-0,43	-0,78	-0,69	-0,55	putative nucleotide-binding protein 8535532:8536947 forward MW:51748	39
SCO7701		-1,02	-0,20	-0,82	-0,65	-0,90	putative methyltransferase 8538368:8539246 forward MW:32792	
SCO7717		-0,93	-0,33	-0,24	-0,29	-1,36	putative secreted protein 8552007:8552420 reverse MW:13763	
SCO7800		0,06	0,56	0,16	1,10	-0,97	hypothetical protein 8621285:8621500 forward MW:7714	
SCO7801		-0,41	0,81	0,27	0,80	-1,07	putative membrane protein 8621520:8621900 forward MW:13812	
SCO7805		-0,48	-0,46	-0,64	-1,11	-0,74	hypothetical protein SC8E7.02. 8625511:8625846 forward MW:11199	
SCO7808		-0,31	-0,67	-1,28	-0,67	-0,26	hypothetical protein SC8E7.05c. 8627358:8627651 reverse MW:10235	

Relevant DasR target genes with fold change <2

SCO1390	*crr*	0,52	0,01	0,18	0,36	0,45
SCO1391	*ptsI*	0,23	0,09	0,17	0,26	0,07
SCO2198	*glnA*	0,75	0,17	0,15	0,62	0,19
SCO2906	*nagE1*	0,43	0,16	0,45	0,69	0,36

SCO4159	*glnR*	0,07	-0,11	0,19	0,13	0,57
SCO4240	*msiK*	-0,02	0,54	0,39	0,93	0,03
SCO4284	*nagA*	0,43	0,21	0,36	0,32	0,44
SCO5777	*gluA*	0,80	0,77	0,98	-0,58	-0,08
SCO3034	*whiB*	-0,17	-0,57	-0,47	-0,47	-0,63
SCO3158	*ssgE*	-0,19	-0,33	-0,14	-0,52	-0,62
SCO3571	*crp*	0,17	-0,01	0,19	0,73	0,78
SCO4768	*bldM*	0,28	0,14	0,17	0,18	0,92
SCO5046	*wblI*	-0,14	-0,31	-0,50	-0,47	-0,77
SCO5114	*bldKc*	0,11	0,30	0,35	0,57	0,72
SCO5115	*bldKd*	0,02	0,40	0,53	0,49	0,47
SCO5116	*bldKe*	0,25	0,64	0,80	0,84	0,44
SCO5320	*whiE-ORFI*	-0,12	-0,42	-0,61	-0,45	0,48
SCO5723	*bldB*	0,32	-0,64	-0,20	-0,41	0,19
SCO5877	*redD*	-0,28	0,19	0,80	0,85	0,04
SCO5881	*redZ*	0,62	0,78	0,62	-0,23	0,22
SCO6685	*ramR*	-0,20	-0,29	-0,21	-0,37	-0,30
SCO3264		0,37	0,50	0,52	0,78	0,43
